# Probiotics *Lactobacillus reuteri* Abrogates Immune Checkpoint Blockade-Associated Colitis by Inhibiting Group 3 Innate Lymphoid Cells

**DOI:** 10.3389/fimmu.2019.01235

**Published:** 2019-06-04

**Authors:** Tingting Wang, Naisheng Zheng, Qin Luo, Li Jiang, Baokun He, Xiangliang Yuan, Lisong Shen

**Affiliations:** ^1^Department of Clinical Laboratory, Xinhua Hospital, Shanghai Jiao Tong University School of Medicine, Shanghai, China; ^2^Department of Gynecology and Obstetrics, Xinhua Hospital, Shanghai Jiao Tong University School of Medicine, Shanghai, China; ^3^Department of Gastroenterology, Shanghai General Hospital, Shanghai Jiao Tong University School of Medicine, Shanghai, China; ^4^Shanghai Key Laboratory of Pancreatic Disease, Shanghai General Hospital, Shanghai Jiao Tong University School of Medicine, Shanghai, China

**Keywords:** immune checkpoint blockade, colitis, gut microbiome, inflammation, innate lymphoid cells

## Abstract

Immune checkpoint blockade (ICB) immunotherapy increases antitumor immunity by blocking cytotoxic-T-lymphocyte-associated protein 4 (CTLA-4) or programmed cell death protein 1 (PD-1)/programmed death-ligand 1 (PD-L1) and displays robust clinical responses in various cancers. However, ICB immunotherapy also triggers severe inflammatory side effects, known as immune-related adverse effects (irAEs). One of the most common toxicities is immune checkpoint blockade-associated colitis (ICB associated colitis). The exact mechanism of ICB associated colitis remains to be explored. Here, we combined ICB (anti–CTLA-4 and anti-PD-1) treatment with a standard colitis model, in which a more severe form of colitis is induced in mice, to recapitulate the clinical observations in patients receiving combined ipilimumab (anti-CTLA-4) and nivolumab (anti-PD-1) therapy, during which colitis is the most frequent complication encountered. We found that the composition of the gut microbiota changed in ICB associated colitis. Principal component analysis of the gut microbiome showed an obvious reduction in the abundance of *Lactobacillus* in severe ICB associated colitis. Lactobacillus depletion completely by vancomycin augmented the immunopathology of ICB. Furthermore, we found that the ICB toxicity could be totally eliminated via the administration of a widely available probiotic *Lactobacillus reuteri (L.reuteri)*. Oral administration of *L. reuteri* therapeutically inhibited the development and progression of colitis, thus ameliorating the loss of body weight and inflammatory status induced by ICB treatment. Mechanistically, the protective effect of *L. reuteri* was associated with a decrease in the distribution of group 3 innate lymphocytes (ILC3s) induced by ICB associated colitis. In conclusion, our study highlights the immunomodulatory mechanism of the gut microbiota and suggests that manipulating the gut microbiota by administrating *L. reuteri* can mitigate the autoimmunity induced by ICB, thus allowing ICB immunotherapy to stimulate the desired immune response without an apparent immunopathology.

## Introduction

Immune checkpoint blockade (ICB) immunotherapy targeting intrinsic downregulators of immunity, such as cytotoxic T-lymphocyte-associated antigen 4 (CTLA-4), programmed cell death protein 1 (PD-1), and programmed cell death ligand 1 (PD-L1) has shown durable clinical responses and recently been a source of promising new cancer treatments (1, 2). ICB Immunotherapy has transformed the oncology field, improving survival in patients suffering from a range of cancer types. To date, several immune-checkpoint-blocking mAbs have been approved for the treatment of patients with several types of cancer, and more and more patients will benefit from ICB immunotherapy in the months and years ahead.

While ICB immunotherapy stimulates T-cell activation and effective antitumor immune responses, it can also have serious inflammatory side effects in some patients, known as immune-related adverse effects (irAEs), which resemble autoimmune disease (3). irAEs are frequent and might occur in up to 90% of patients treated with the anti-CTLA-4 antibody (4) and in 70% of patients treated with a PD-1/PD-L1 antibody (5). Although any organ system can be affected, irAEs most commonly involve the gastrointestinal tract, endocrine glands, skin, and liver (6). One of the most common toxicities is immune checkpoint blockade-associated colitis (ICB associated colitis) (7). ICB associated colitis can be quite severe and represents a distinct form of colitis with characteristics reminiscent of inflammatory bowel disease (IBD) (8, 9). Clinically, ICB-associated colitis is routinely treated with immunosuppressive therapy, which diminishes the antitumor response of ICB and has significant side effects (10). Recommendations regarding the optimal management of ICB associated colitis need to be evolved (10, 11). Recently, several reports from cohorts of ICB-treated patients suffering from colitis have confirmed the similarities between ICB-associated colitis and IBD both clinically and endoscopically (8). There is significant overlap between the conditions, including the development of deep ulceration, a negative prognostic factor. Histologically, ICB-associated colitis exhibits features of chronic damage, including IBD- and lymphocytic colitis-like phenotypes. The immunopathogenesis of ICB-associated colitis appears to be predominantly associated with mucosal Th1/Th17 effector responses (12). However, the exact mechanism underlying ICB-associated colitis remains to be identified. Uncovering the mechanisms involved in its pathogenesis will greatly enhance our understanding and therapeutic management of ICB-mediated colitis.

Recently, several studies have started to highlight the crucial role of the gut microbiota in the antitumor responses induced by checkpoint blockade antibodies (6, 7). The composition of the gut microbiota has been shown to affect antitumor immune responses (13–17). A rapidly expanding concept is that the gut microbiome affects not only the efficacy of the treatment but also the toxicity due to ICB. Indeed, novel evidence supports the idea that in both anti-CTLA-4 and anti-PD-1 colitis, specific “favorable” or “unfavorable” microbiome profiles may affect the efficacy of the immune response and, consequently, the appearance of gastrointestinal irAEs (18, 19). The syndromes of irAEs mostly frequently affect the gastrointestinal tract and the skin which exposed to commensal microorganisms. However, a knowledge gap remains regarding how the gut microbiota influences gastrointestinal irAEs. *L. reuteri* are Gram-positive, rod-shaped, and anaerobic. As a common bacterial strain coexisting in human and animal gastrointestinal tract, *L. reuteri* has been confirmed that have many excellent probiotic characteristics. First, LR secretes antibacterial substances such as lactic acid, hydrogen peroxide to regulate intestinal pH and microenvironment to inhibit the colonization of pathogenic microbes and remodel the commensal microbiota (20). A recent study indicated that *L. reuteri* can also induce anti-inflammatory Treg cells, and mediate suppression of Th1/Th2 responses (21). Also, bearing the ability to strengthen the intestinal barrier, the colonization of *L. reuteri* may decrease the inflammation in the gut. It has been confirmed by several studies that *L. reuteri* can alleviate DSS induced colitis by inhibiting proinflammatory gene expression (22) and reducing P-selectin-associated leukocyte- and platelet-endothelial cell interactions (23). However, the effect of *L. reuteri* on the appearance of gastrointestinal irAEs is underexplored.

In the present study, we established a dextran sulfate sodium (DSS)-induced colitis and B16 melanoma tumor mouse model to imitate the clinical outcomes of patients receiving ipilimumab (Anti-CTLA-4) and nivolumab (anti-PD-1), for whom colitis is the most frequent problem encountered. We conducted this model to study the impact of the composition of the gut microbiota on the immunopathology of ICB-associated colitis, and explore the therapeutic way to mitigate ICB-induced autoimmunity by manipulating the gut microbiota to allow checkpoint blockade to achieve the desired immune response.

## Materials and Methods

### Mouse Strains

C57BL/6 mice were purchased from SLAC Laboratory Animals Co., Ltd. (Shanghai, China). For all of the experiments, 8 weeks old female mice were used. The mice were maintained in the Shanghai Laboratory Animal Center of China. The mouse experiments were approved by the Ethics Committee of Xinhua Hospital, Shanghai Jiao Tong University School of Medicine.

### Cell Lines

The B16 cell line was purchased from Shanghai Institutes for Biological Sciences (Shanghai, China), and the cells were cultured in RPIM Medium 1640 (Gibco, Life Technologies, USA) containing 10% FBS (Gibco, USA), 100 U/mL penicillin, and 100 μg/mL streptomycin, at 37°C in a humidified atmosphere of 5% CO_2_.

### Generation of Inflammation Mouse Models

The mice received 3% DSS (MP Biomedicals) in their drinking water for 10–15 d. Weight was recorded daily. For the gut commensal manipulation, mice were treated with vancomycin (0.5 g/L, Sigma, USA) in the drinking water for at least 14 d. Afterward, DSS was added to their drinking water. For ICB-associated colitis, The mice were injected once every other day (initiated 3 day before the DSS administration) with 100 μg of anti-CTLA-4 mAb (Bioxcell, USA) and 250 μg of anti-PD-1 mAb (Bioxcell, USA) or isotype control antibody before the DSS administration.

### Tumor Challenges and Animal Treatment

2 × 10^5^ B16 tumor cells were subcutaneously (s.c.) injected into the right flanks of the mice. The mice were injected intraperitoneally (i.p.) with 100 μg of anti-CTLA-4 mAb and 250 μg of anti-PD-1 four times after 7, 10, 13, and 16 days of tumor incubation. Tumor size was measured with a caliper and calculated as the length × width × width × 0.5236.

### *L. Reuteri* Administration

*L. reuteri ATCC PTA 6475* cells resuspended in PBS were used in this study. Each mouse was given 300 μL of *L. reuteri* (1 × 10^9^ CFU per mouse) via oral gavage. In the DSS colitis and tumor model, *L. reuteri* was administered daily before the DSS administration or tumor injection for 3 days until the samples collected.

### Tissue Isolation and Flow Cytometry Assay

For tumor isolation, B16 tumor explants were removed after euthanizing the mice. The isolated tumors were cut into small pieces with scissors. Next, the pieces were digested with collagenase Type IV (1 mg/mL; Worthington, USA), DNase I (0.02 mg/mL; Sigma-Aldrich, USA), hyaluronidase (0.1 mg/mL; BBI Life Sciences, USA) at 37°C for 30 min. The tumor samples were then pressed through 70 μm nylon filters (BD Biosciences, USA) to create single-cell suspensions. For the colon isolation: The colon was carefully dislodged, while simultaneously removing the mesenteric fat. The caecum was removed, and the colons were placed into cold PBS to remove most of the feces. The samples were incubated in Hank's balanced salt solution with 5 mM EDTA (Invitrogen, USA) and without calcium or magnesium (Beyotime, China) at 37°C with agitation (120 rpm) three times for 20 min each time. Scissors were used to cut the gut into small pieces, and the pieces were digested in Hank's balanced salt solution with calcium and magnesium (Beyotime, China) supplemented with collagenase Type IV (1 mg/mL; Worthington, USA), DNase I (500 μg/mL; Sigma-Aldrich, USA), dispase (200 μg/mL; Sigma, USA), and 2% FCS at 37°C for 20 min. The samples were then pressed through 70 μm nylon filters (BD Biosciences, USA). The digestion was repeated three times, and 40 and 80% Percoll solutions were used (Yeasen, China) to disrupt the cells to create single-cell suspensions. For the FACS analysis, the cells were stained with antibodies to the following markers: CD45 (clone 30-F11), CD3 (clone 17A2), CD3 (clone 145-2C11), CD4 (clone GK1.5), CD8a (clone 53-6.7), GR-1 (clone 1A8), CD19 (clone 1D3), CD127 (IL7Ra) (clone A7R34), RORγt (clone Q31-378), NKp46 (clone 29A1.4), KLRG-1 (clone 2F1/KLRG1), CD11b (clone M1/70), Ly-6G (clone RB6–8C5), ki67 (clone SolA15). All of the antibodies were produced by BD, eBioscience, or Biolegend and are listed in Supplementary Table S1. Dead cells were excluded from the analysis using Fixable Viability Dye eFluor 450 (eBioscience, USA). For the intranuclear staining, the cells were fixed and permeabilized using the Foxp3 staining kit (eBioscience, USA). All of the flow experiments were performed on FACS Canto II machines (BD, USA), and the flow cytometry data were analyzed with FlowJo 10.

### Colitis Scores and Histologic Analysis

Freshly isolated colons were fixed in formalin and embedded in paraffin. H&E staining was performed using a standard protocol. For the quantitative histological analysis, five criteria were used to grade each section of the intestine: (i) severity of inflammation, (ii) percent of area affected by inflammation, (iii) degree of hyperplasia, (iv) depth of the lesion, and (v) ulceration.

### 16S rRNA Gene Sequencing and Analyses

The genomic DNA contained in the stool samples was extracted using an extraction kit (E.Z.N.ATM Mag-Bind Soil DNA Kit, Omega, USA) according to the manufacturer's instructions. We measured the concentration of the DNA using a Qubit 2.0 (life, USA) to ensure that adequate amounts of high-quality genomic DNA had been extracted and the DNA quality assessed using a bioanalyzer (Agilent 2100, USA). The PCR was performed immediately after the DNA was extracted. The V3-V4 region of the bacterial 16S rRNA gene was amplified using the universal primers (24, 25) 341F (CCTACGGGNGGCWGCAG) and 805R (GACTACHVGGGTATCTAATCC). The reaction was set up as follows: microbial DNA (10 ng/μL) 2 μL; amplicon PCR forward primer (10 μM) 1 μL; amplicon PCR reverse primer (10 μM) 1 μL; 2 × KAPA HiFi Hot Start Ready Mix 15 μL (total 30 μL). The plate was sealed and PCR performed in a thermal instrument (Applied Biosystems 9700, USA) using the following program: 1 cycle of denaturing at 95°C for 3 min, first 5 cycles of denaturing at 95°C for 30 s, annealing at 45°C for 30 s, elongation at 72°C for 30 s, then 20 cycles of denaturing at 95°C for 30 s, annealing at 55°C for 30 s, elongation at 72°C for 30 s and a final extension at 72°C for 5 min. The PCR products were checked using electrophoresis in 1% (w/v) agarose gels in TBE buffer (Tris, boric acid, EDTA) stained with ethidium bromide (EB) and visualized under UV light. The PCR products were checked using electrophoresis in 1% (w/v) agarose gels in TBE buffer (Tris, boric acid, EDTA) stained with ethidium bromide (EB) and visualized under UV light. We used AMPure XP beads to purify the free primers and primer dimer species in the amplicon product. Barcodes unique to each sample were incorporated at the beginning of the forward primers, which allowed the identification of each sample in a mixture during an Illumina sequencing run. The sequencing was performed using the Illumina MiSeq system (Illumina MiSeq, USA) according to the manufacturer's instructions. The rarefaction curves and the diversity indices were determined based on the calculated operational taxonomic units (OTUs) by Mothur ver. 1.30.1. The obtained sequences were phylogenetically sorted to the phylum, class and genus levels at 97% similarity for the community composition analysis. For the taxonomic analysis, the representative sequences from each OTU were subjected to the RDP-II Classifier of the Ribosomal Database Project (RDP) and the BLAST algorithm of the National Center for Biotechnology Information (NCBI). The 16S rRNA sequence data have been deposited in the NCBI BioProject with accession number PRJNA525923.

### Serum Cytokine Analysis

Blood samples were collected on one time point on day 10 (DSS + ICB + Van group) or day 15 (DSS group/ICB+DSS group/ *L.reuteri group*) days according to the weight loss of mice after colitis induction with DSS. After clotting for at least 30 min at room temperature, the plasma was separated in a centrifuge (10 min at 1,500 relative centrifugal force). The secreted plasma levels of a range of inflammatory cytokines (CXCL1, TNF-α, IL-12 p70, IL-1β, IL-2, IL-4, IL-6, IL-10, IL-13, IFN-γ) were assessed using a mouse Magnetic Luminex Assay Kit (R&D Systems, USA) and quantified using a Luminex multiplexing suspension array system (LUMINEX X-200, R&D, USA) following the manufacturer's instructions. The cytokines of IL-23, IL-22, and IL-17 in intestine tissues and sera of mice were assessed using the mouse ELISA Assay Kits (R&D Systems, USA) following the manufacturer's instructions.

### Statistical Analysis

In each experiment, multiple mice were analyzed as biological replicates. All of the statistical analyses were performed using GraphPad Prism 7.0 software and displayed as the mean ± SEM. The statistical significance of any differences was assessed using Student's *t*-test, and one-way ANOVA was conducted for multiple comparisons. For the survival analysis, Kaplan-Meier survival curves were calculated, and the statistical significance was determined by the log-rank test. In the analysis of the component differences between groups, Welch's *t*-test and one-way ANOVA with the Tukey-Kramer *post-hoc* test were used via STAMP. The eta-squared (η2) value was used to estimate the effect size. Differences were considered statistically significant when the *p*-value was <0.05, and the significance levels are represented by ^*^P < 0.05, ^**^P < 0.01, ^***^P < 0.001, and ^****^P < 0.0001, unless denoted otherwise. The 16S rRNA sequence data have been deposited in the NCBI BioProject with accession number PRJNA525923.

## Results

### ICB Treatment Increased the Susceptibility of DSS-Induced Colitis in ICB Receiving Mice

**Figure 1:**
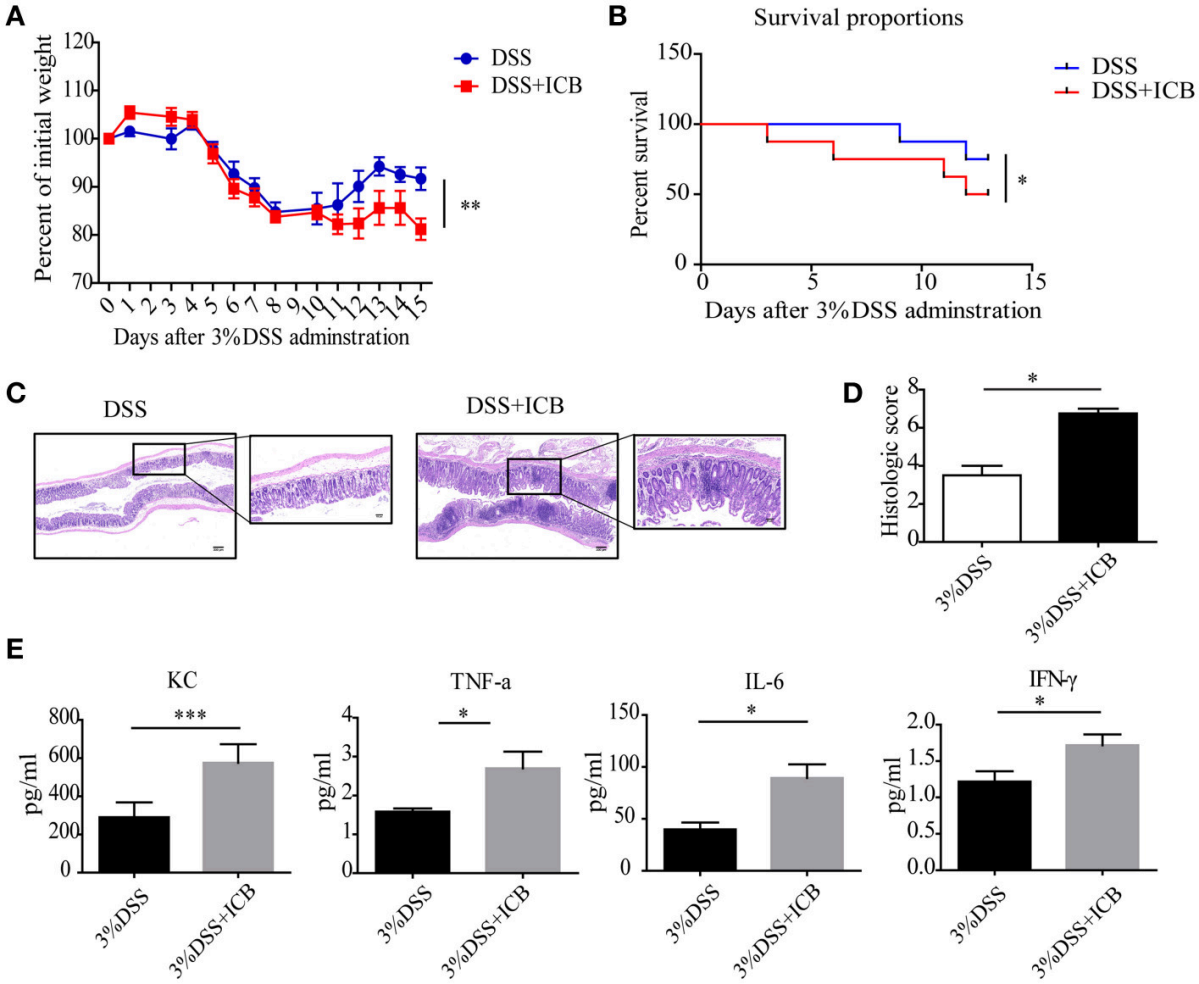
Increased susceptibility to DSS-induced colitis in ICB-receiving mice. **(A)** Weight loss curves of mice treated with 3% DSS and receiving the ICB (ICB: αCTLA-4 mAb and αPD-1 mAb) or IgG isotype control (Iso Ctrl). Mice were given 3% DSS for 7 d. *n* = 10 per group. **(B)** Survival curves (in percent) of the mice receiving the IgG isotype control (Iso Ctrl) or the ICB treatment along with 3% DSS administration. Survival was monitored for 14 d, *n* = 10 per group. **(C)** Representative colon histological results from mice treated with an injection of the isotype control (left) or ICB treatment (right) along with 3% DSS administration. Colon samples were collected on day 15 and stained with H&E (Scale bar, 200 μm). **(D)** The colon histological scores of mice receiving the IgG isotype control (Iso Ctrl) or the ICB treatment along with 3% DSS administration, *n* = 5 per group. **(E)** The KC, TNF-α, IL-6, and IFN-γ concentrations in the sera of mice treated with isotype control (Iso Ctrl) or the ICB treatment along with 3% DSS administration, *n* = 5 per group. Means with SEM analyzed by unpaired Student's *t*-test. *P < 0.05, **P < 0.01, and ***P < 0.001.

Clinically, immune checkpoint blockade (ICB)-associated colitis closely resembles the colitis associated with IBD pathophysiology (8). To establish a checkpoint blockade-related autoimmune mouse model, mice were tested to determine their response to orally administered dextran sulfate sodium (DSS) with an injection of a combination of anti-CTLA-4 and anti-PD-1 antibodies or isotype control. After administration of 3% DSS via the drinking water for 7 days, the mice that received ICB treatment showed more severe weight loss compared to that of mice that received the isotype control antibody (Figures 1A,B). There was significantly efficacy without significant weight loss in ICB treated mice in the absence of DSS (Supplementary Figures S1, S2). Histological colonic sections from the ICB associated mice model (Supplementary Figure S3A) showed that the animals that received the ICB combined treatment exhibited more severe exacerbated hyperplasia, more inflammatory leukocyte infiltration, and severe ulceration compared with the mice that received DSS without ICB treatment but isotype control antibody, resulting in poorer histopathological scores (Figures 1C,D). The serum levels of four inflammatory cytokines, KC, TNF-α, IL-6, and IFN-γ were dramatically increased in the ICB-treated mice (Figure 1E, Supplementary Figure S3B). Furthermore, we observed a therapeutic effect of the combination treatment with anti-CTLA-4 and anti-PD-1 antibody against established B16 melanomas in the same mice (Supplementary Figures S1A–C), as reported previously. Thus, the data from our B16 tumor model and DSS-induced colitis are consistent with clinical observations in patients who received ipilimumab (anti-CTLA-4 antibody), and/or nivolumab (anti-PD-1 antibody), for whom colitis is the most commonly encountered problem.

### The Immunopathology of ICB Associated Colitis Impacts the Gut Microbiota

**Figure 2:**
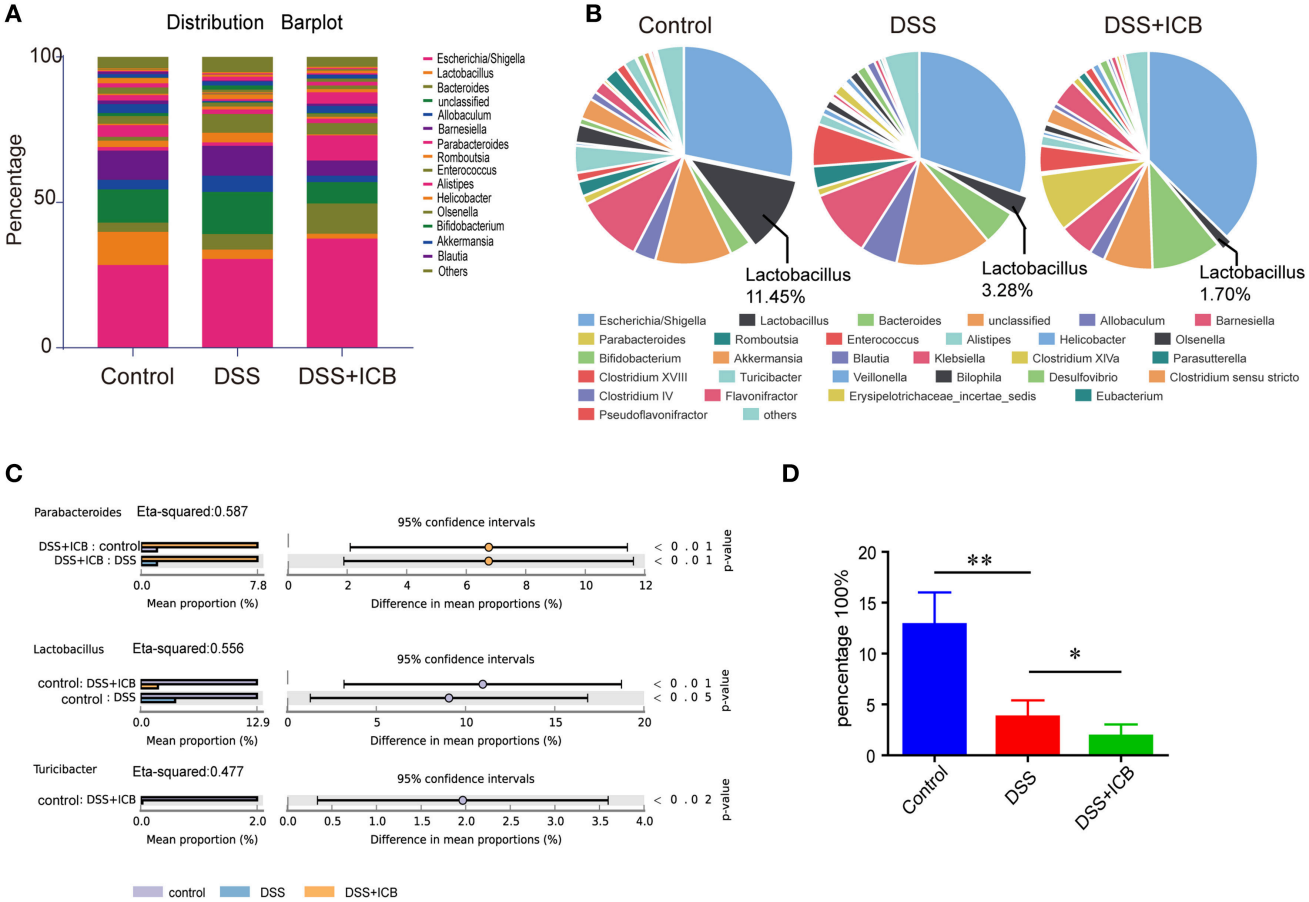
Microbiome analysis of the intestinal bacteria in DSS-treated mice receiving ICB treatment as assessed via 16S rRNA gene amplicon sequencing. **(A)** Microbial community bar plot sorted by genus for the mice receiving the IgG isotype control (Iso Ctrl) or the ICB treatment along with 3% DSS administration. Relative abundances of the predominant bacteria (>1% in any sample) in the feces of mice receiving the IgG isotype control (Iso Ctrl) or the ICB treatment along with 3% DSS administration. *n* = 5 per group. **(B)** Pie chart showing the relative abundances of the predominant bacteria (>1% in any sample) in the feces of mice treated with an injection of isotype control (Left) or ICB treatment (Right) along with 3% DSS administration. *n* = 5 per group. **(C)** different abundance analysis of the top 3 enriched predominant bacteria in mice treated with an injection of isotype control (Left) or ICB treatment (Right) along with 3% DSS administration. **(D)** Relative abundance of *Lactobacilli* in the feces of mice receiving the IgG isotype control (Iso Ctrl) or the ICB treatment along with 3% DSS administration. *n* = 5 per group. *P < 0.05 and **P < 0.01.

Several lines of evidence support a critical role for the microbiota in the experimental colitis system (26). To determine the changes in the commensal bacteria composition with the development of ICB related colitis, fecal samples collected from mice receiving the IgG isotype control (Iso Ctrl) or the ICB treatment along with 3% DSS administration were submitted for bacterial microbiota profiling using 16S ribosomal RNA sequencing on the Illumina MiSeq platform. The histograms in Figure 2A illustrate the gut microbiota community structures and reveal the microbial species and their relative abundances. As shown in Figure 2B, all of the samples contained *Escherichia, Lactobacillus, Bacteroides, Allobaculum, Barnesiella*, and *Parabacteroides*. Analysis of the results showed that the relative abundances of *Parabacteroides, Lactobacillus*, and *Turicibacter* were significantly different among the three groups (Figure 2C). The most abundant phyla were *Escherichia, Allobaculum*, and *Lactobacillus*, and the least abundant phylum was *Lactobacillus* in the mice that received ICB treatment with 3% DSS administration (Figures 2C,D). Hierarchical clustering of the samples based on the relative abundance of the bacterial species also revealed that the relative population of *Lactobacillus* was significantly lower in the stool of ICB-treated mice compared to that in the stool from mice in the control and DSS administration only groups (*P* < 0.01; Figure 2D).

### Depletion of Lactobacillus by Vancomycin Augments the Immunopathology of ICB

**Figure 3:**
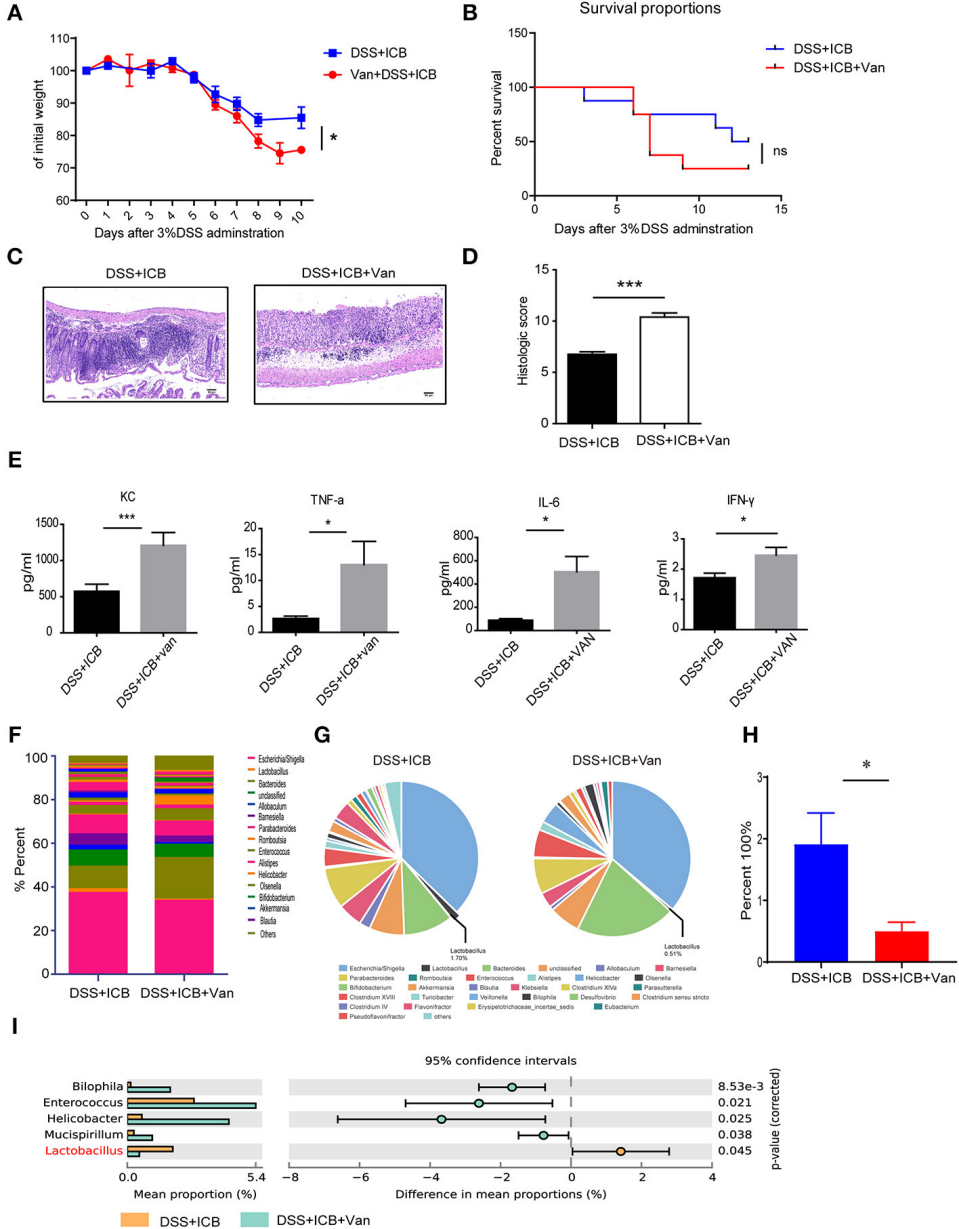
Vancomycin augments the immunopathology of ICB in DSS-treated mice. **(A)** Weight loss curves of water- or vancomycin-treated mice receiving the ICB (ICB: αCTLA-4 mAb and αPD-1 mAb) along with the administration of 3% DSS. Mice were given 3% DSS for 7 d. *n* = 15 per group. **(B)**, Percent survival curves of water- (left) or vancomycin-treated mice (right) receiving the ICB treatment along with 3% DSS administration. Survival was monitored for 14 d, *n* = 15 per group. **(C)**, Representative colon histology results from water- (left) or vancomycin-treated mice treated with an injection of isotype control or ICB treatment (right) along with 3% DSS administration. Colon samples were collected on day 10 and H&E stained (Scale bar, 50 μm). **(D)** The colon histological scores of water- or vancomycin-treated mice receiving the ICB treatment along with 3% DSS administration, *n* = 5 per group. **(E)** The concentrations of KC, TNF-a, IL-6, and IFN-γ in the sera of water- or vancomycin-treated mice with the indicated treatments. *n* = 5 per group. **(F)** Microbial community bar plot sorted by the genus of the mice receiving ICB treatment and 3% DSS administration with or without vancomycin. The relative abundances of the predominant bacteria (>1% in any sample) in the feces of mice receiving the ICB treatment along with 3% DSS administration. *n* = 5 per group. **(G)** Pie chart shows the relative abundances of the predominant bacteria (>1% in any sample) in the feces of mice treated with ICB treatment and 3% DSS administration with or without vancomycin. *n* = 5 per group. **(H)** Relative abundances of *Lactobacilli* in the feces of mice receiving ICB treatment and 3% DSS administration with or without vancomycin. *n* = 5 per group. **(I)** the comparison of the top 3 enriched predominant bacteria in mice treated with an injection of ICB and 3% DSS administration with or without vancomycin. *n* = 5 per group. Means with SEM analyzed by unpaired Student's *t*-test. *P < 0.05 and ***P < 0.001.

*Lactobacillus* is a genus of Gram-positive anaerobic bacteria. We wondered if depletion of *Lactobacillus* by vancomycin (an antibiotic activity against Gram-positive bacteria, including lactobacillus) would impact the severity of ICB-associated colitis. Previously, a study showed that vancomycin exacerbates histopathological signs of gut inflammation (16). To test whether the loss of *Lactobacillus* or other strains contributed to the severe colitis, we assessed the impact of vancomycin on the colitis severity after ICB treatment in the DSS colitis model. The mice were pretreated with vancomycin for 2 weeks before the induction of colitis. In the vancomycin group, the mice started to show lower body weight from 6 d of DSS with ICB treatment (Figure 3A), showing more severe colitis that occurs in the absence of antibiotic treatment. Consistently, more severe weight loss was observed in mice pretreated with vancomycin than in mice that received the water control (Figure 3A). By day 9, 80% of the vancomycin plus DSS and ICB-treated mice had died, whereas only 20% of mice given the water control had died (Figure 3B). The histopathological scores were also significantly worse for the vancomycin-treated mice than for the controls (Figure 3C). H&E staining of colon sections showed complete ulceration and severe immune cell infiltration in the vancomycin-treated mice (Figure 3D). Similar serum levels of three inflammatory cytokines, KC, TNF-a, IL-6, and IFN-γ, were also dramatically increased in the vancomycin-treated mice (Figure 3E and Supplementary Figure S4). Furthermore, stool microbiome analyses also confirmed that vancomycin administration decreased the *Lactobacillus* abundance to an undetectable level (Figures 3F–I).

### Direct Administration of *L. Reuteri* Ameliorates the Immunopathology of ICB

**Figure 4:**
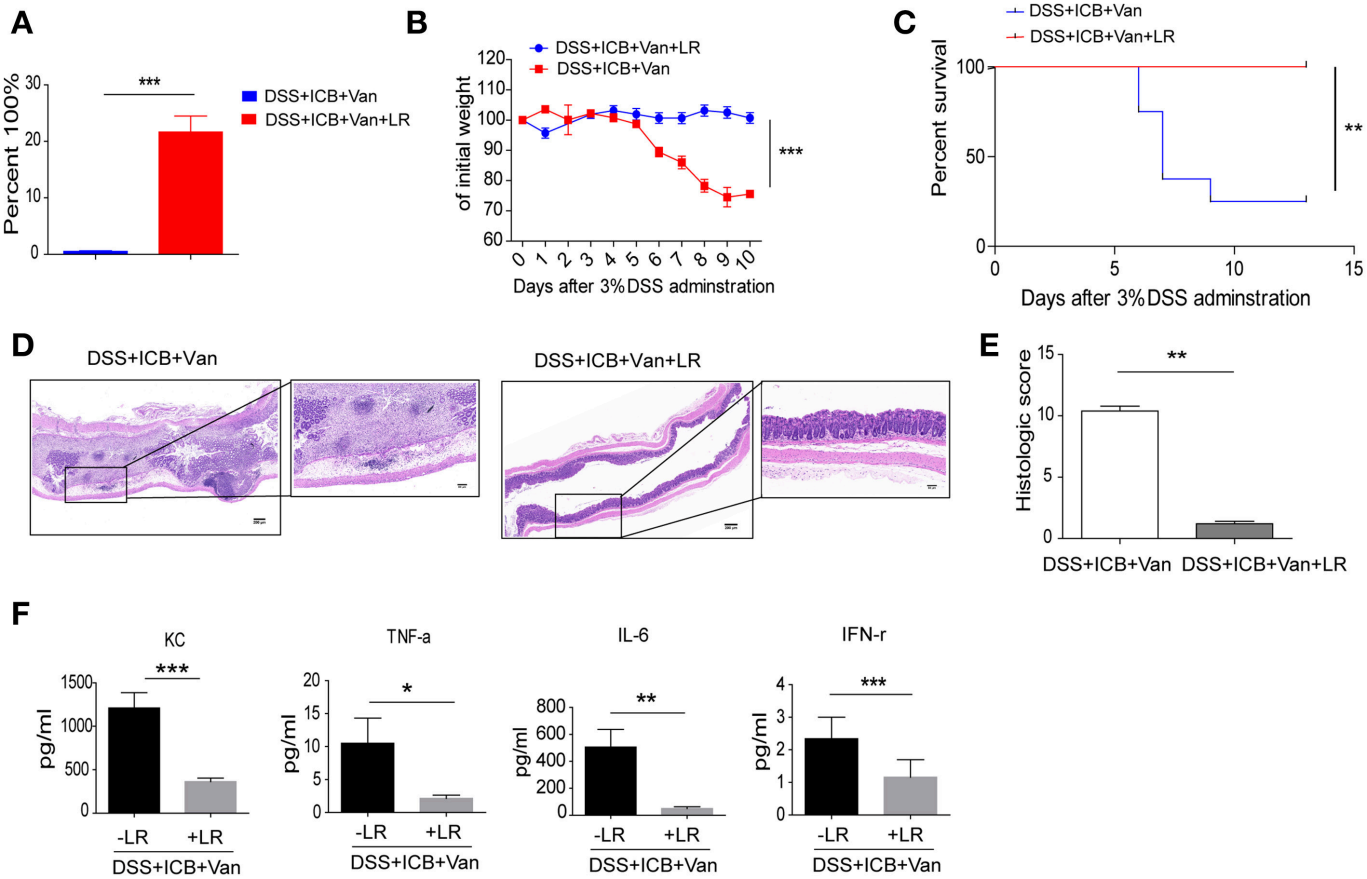
*L. reuteri* therapeutically abrogates ICB-associated intestinal inflammation in DSS-treated mice. **(A)** The relative abundance of *L. reuteri* was quantified with 16s RNA sequencing. This value was normalized to the total bacteria, *n* = 5 per group. ***P < 0.001. **(B)** Weight loss curves of ICB-injected mice with 3% DSS-induced colitis treated either with vancomycin + PBS, or vancomycin + *L. reuteri, n* = 15 per group. **(C)** Percent survival curves of in ICB-injected mice with 3% DSS-induced colitis treated as described in A. Survival was monitored for 14 d, *n* = 15 per group. **(D)** Representative colonic histology results from ICB-injected mice with 3% DSS-induced colitis with the indicated treatments. Colon samples were collected on day 10 and H&E stained (Scale bar, 200 μm) **(E)** Quantification of the colon histological scores of ICB-injected mice with 3% DSS-induced colitis treated as described in A, *n* = 5 per group. **(F)** Serum concentrations of KC, TNF-α, IL-6, and IFN-γ in the serum of in ICB-injected mice with 3% DSS-induced colitis with the indicated treatments, *n* = 5 per group. Means with SEM analyzed by unpaired Student's *t*-test. *P < 0.05, **P < 0.01, and ***P < 0.001.

To directly test the protective effect of the probiotic Lactobacillus in this colitis model, we obtained a commercially available probiotic *L. reuteri* and administered the bacteria to the mice via oral gavage before the induction of DSS colitis (Supplementary Figure S5A). This probiotic *L. reuteri* treatment resulted in a 20-fold increase in the relative abundance of these bacteria in the feces (Figure 4A and Supplementary Figures S5B–D). Under our combined ICB conditions, *L. reuteri* treatment improved ICB induced colitis (Supplementary Figures S6A–E). *L. reuteri* administration completely rescued the severe weight loss induced by vancomycin and ICB treatment in the DSS colitis model mice (Figures 4B,C). The average weight of the vancomycin-treated mice was improved from 70% of their initial weight in the DSS plus ICB group to almost 100% in the *L. reuteri* group on day 10 after DSS administration (Figure 4B). Mice treated with both *L. reuteri* and vancomycin exhibited no weight loss (Figure 4B), suggesting that *L. reuteri* treatment ameliorated the immunopathology associated with ICB by helping to rescue the vancomycin-induced gut dysbiosis. Consistent with this assessment, H&E staining of colon sections also revealed that *L. reuteri* treatment resulted in a reduced histopathological score with partial restoration of the colon structure and less leukocyte infiltration in gut tissue (Figures 4D,E). Similarly, *L. reuteri* treatment also decreased the serum levels of the inflammatory cytokines KC, TNF-a, IFN-γ, and IL-6 in the DSS colitis mice (Figure 4F and Supplementary Figure S7). Taken together, our results demonstrate that coadministration of the probiotic *L. reuteri* could rescue the mice from the immunopathology induced by ICB-associated colitis.

### *L. Reuteri* Does Not Affect Antitumor Immunity of ICB

**Figure 5:**
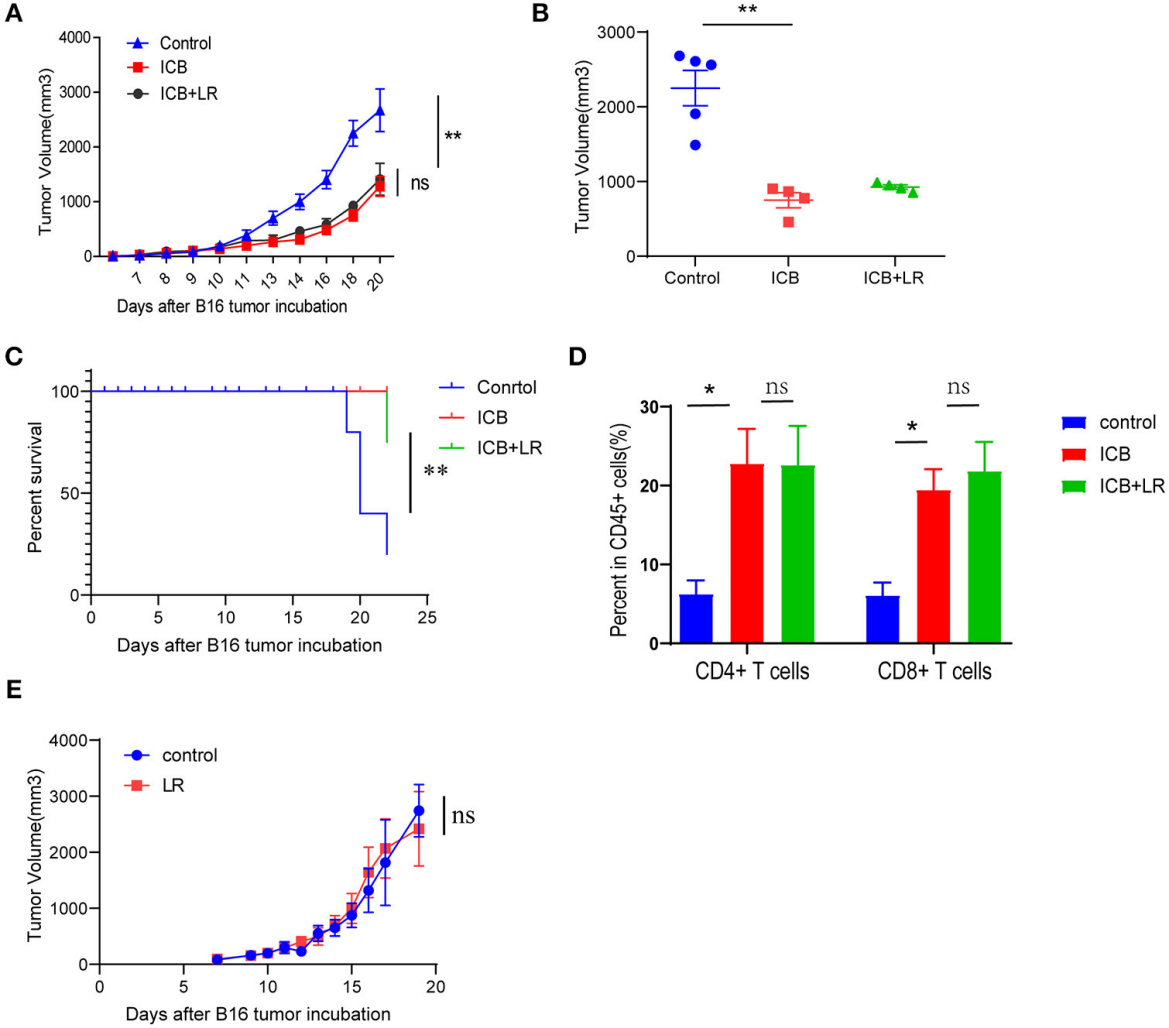
Biological affection of *L. reuteri* during anticancer ICB treatment. **(A)** B16 tumor growth kinetics in mice treated with PBS or *L. reuteri* by oral gavage, followed by treatment with Ctrl or ICB. The αCTLA-4 and αPD-1 mAb were injected at 7, 10, 13, and 16 d post-tumor implantation, *n* = 5 per group. The data are reported as the means and the error bars represent the SEMs. **(B)** Tumor sizes of the B16 tumors on day 19 post implantation in mice treated with PBS or *L. reuteri* by oral gavage, followed by treatment with Ctrl or ICB. **(C)** Percent survival curves of B16 tumor-bearing mice treated with PBS or *L. reuteri* by oral gavage, followed by treatment with Ctrl or ICB. **(D)** Quantification of intratumoral CD4^+^ T cells and CD8^+^ T cells in B16 melanoma tumor by flow cytometry (18 days of post tumor implantation; *n* = 5 per group). **(E)** tumor growth kinetics in mice bearing B16 melanoma treated with PBS or *L. reuteri* by oral gavage, *n* = 5 per group. Means with SEM analyzed by unpaired Student's *t*-test. *P < 0.05 and **P < 0.01. n.s., not significant.

We further examined whether the amelioration of the immunopathology by *L. reuteri* occurs at the cost of ICB efficacy. Treatment with the probiotic *L. reuteri* did not affect the growth kinetics of established B16 melanoma tumors with ICB administration (Figure 5A). Tumors of comparable size were found in *L. reuteri*-treated mice and in PBS-treated control mice administrated with ICB treatment on day 19 post-inoculation (Figure 5B). Probiotic *L. reuteri* treatment also did not affect the total survival of mice bearing established B16 melanoma tumors (Figure 5C). Flow data also showed that *L. reuteri* administration was accomplished without significant changes in the tumor-infiltrating T cells, predicting a minor or no effect on the antitumor effect of the immunotherapy (Figure 5D). Moreover, a single *L. reuteri* administration has no effect on the growth kinetics of established B16 melanoma tumors (Figure 5E). These data suggest that *L. reuteri* ameliorated the immunopathology without compromising the therapeutic efficacy of ICB against melanoma in this system.

### The Immune Regulatory Function of *L. Reuteri* Is Associated With Intestinal ILC3 Cells

We next investigated the immunologic mechanism underlying the observed amelioration of colitis in *L. reuteri*-treated mice. To evaluate the effect of *L. reuteri* on the immune system in this DSS colitis model, we analyzed the profile of the immune cells in the gut, and we found that administration of ICB increases the frequency of CD8+ T cells. However, we did not observe any significant differences between PBS- and *L. reuteri*-treated mice with respect to the amounts of CD45^+^ immune cells, myeloid cells or T cells isolated from the colons (Supplementary Figures S8A–D).

**Figure 6:**
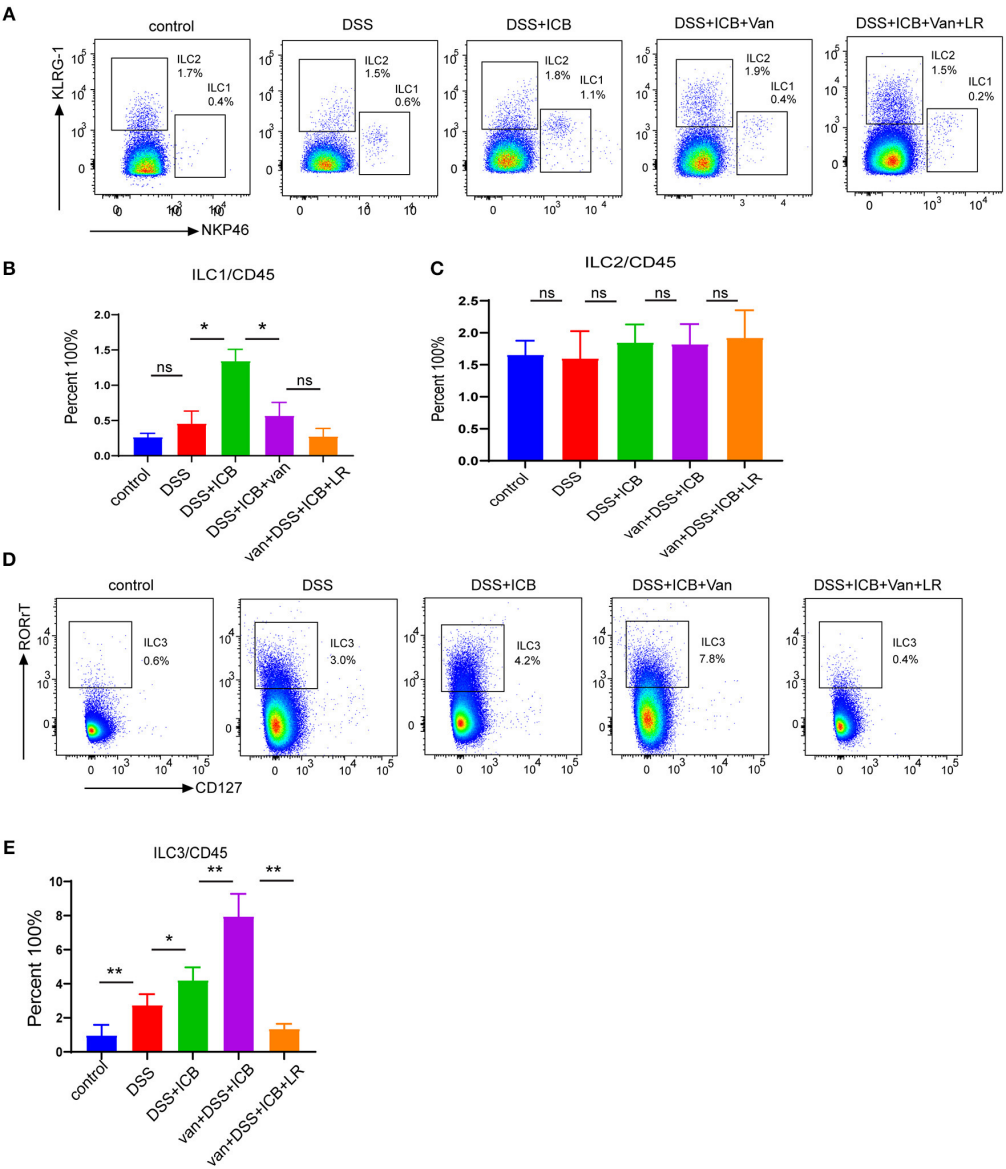
Immunological infiltrate of innate lymphoid cells in ICB-associated intestinal inflammation of DSS-treated mice with or without *L. reuteri* administration. **(A)** Representative color plots showing the flow cytometric analysis of the canonical ILC subtypes (ILC1s and ILC2s) isolated from the large intestinal lamina propria of mice receiving the IgG isotype control (Iso Ctrl), ICB, ICB+Van, and ICB+Van+ *L. reuteri* (LR) treatment along with 3% DSS administration. **(B)** Quantification of the intestine-infiltrated RORrt^−^NKp46^+^ ILC1 populations in the total CD45^+^ immune cells from the indicated groups, *n* = 10 per group. **(C)** Quantification of the intestine-infiltrated RORrt^−^KLRG-1^+^ ILC2 populations in the total innate lymphoid cells from the indicated groups, *n* = 10 per group. **(D)** Representative color plots showing the flow cytometric analysis of the canonical ILC subtype (ILC3s) isolated from the large intestinal lamina propria of mice receiving the indicated treatment. **(E)** Quantification of the intestine-infiltrated RORrt^+^ILC3s cell population in the total CD45^+^ immune cells from the indicated groups, *n* = 10 per group. Means with SEM analyzed by unpaired Student's *t*-test. *P < 0.05, **P < 0.01 and ***P < 0.001.

Recently, a newly identified class of innate lymphoid cells, termed ILCs, has been shown to play a pivotal role in the development of IBD and shaped by microbiome (27–30). To evaluate whether ILCs were involved in the immunological mechanism underlying the observed amelioration of the colitis in *L. reuteri*-treated mice, we first characterized the three canonical subsets of helper-like ILCs. To this end, we used combined biomarkers to analyze the RORgt-NKp46+ ILC1s, RORgt-KLRG-1+ ILC2s, and RORgt+ILC3s by flow cytometry (31) (Supplementary Figure S9). The flow cytometric analysis revealed that although there were no significant changes in the total numbers of CD3^+^ T cells in any of the groups, we did find dramatic differences in the composition of the innate lymphoid cells among the different treatment groups. Interestingly, the magnitudes of the changes in ILC abundance were highly distinct between the subgroups, and the relative changes in the ILC1 and ILC2 subsets within each subgroup were not significant (Figures 6A–C). Of note, the spectrum of the changes in the abundance of the group 3 innate lymphoid cells (ILC3s) corresponded exactly with the severity of the immunopathology associated with DSS colitis (Figure 6D). Group 3 innate lymphoid cells (ILC3s) are an emerging class of innate lymphocytes that play a critical role in regulating mucosal homeostasis. In our DSS colitis model, the severity of that ICB-associated colitis was associated with increased mucosal numbers of ILC3s, but not with changes in the numbers of ILC1s and ILC2s, suggesting that ILC3s are involved in the immunopathology of ICB-associated colitis. *L. reuteri* administration dramatically decreases the mucosal numbers of ILC3s (*P* < 0.01) (Figure 6E) and level of function cytokines IL23 and IL17 (Supplementary Figures S10A–C), which therapeutically prevented colitis in ICB-treated mice (Figure 6D). Thus, it is likely that *L. reuteri* acts on the immunopathology of ICB-associated colitis primarily by affecting the local number of ILC3s.

## Discussion

Despite achieving great clinical success, ICB immunotherapy as a monotherapy or as a part of various combinational strategies has challenges and limitations including the development of irAEs. ICB-associated colitis, one of the irAEs, is quite frequent and severe and is routinely treated with immunosuppressive therapy, which has significant side effects that diminished the antitumor response. Our study demonstrates a role for Lactobacillus in eliminating the intestinal immunopathology associated with ICB associated colitis.

Immune checkpoint inhibition is a recently introduced, innovative form of cancer immunotherapy that aims to eliminate inhibitory costimulatory signals from T cells, mainly tumor-specific cytotoxic CD8+ T cells, via blockade of cytotoxic T-lymphocyte associated protein-4 (CTLA-4), and/or programmed death protein-1 (PD-1)/PD-ligand 1 (PD-L1) (32–34). The treatment consists of administration of monoclonal antibodies that target CTLA-4 (ipilimumab, tremelimumab), PD-1 (pembrolizumab, nivolumab), and PD-L1 (atezolizumab, avelumab, durvalumab) to restore the cytotoxic function of lymphocytes and to induce effective antineoplastic responses (35). In contrast, elimination of immunoregulatory control by T cell-dependent inhibitory pathways may lead to unrestrained activation of effector immune responses that ultimately result in irAEs, which can involve several organs and lead to serious damage. Gastrointestinal toxicity is among the most common and potentially serious irAE and the most frequent reason for discontinuing immunotherapy. Overt colonic inflammation after anti-CTLA-4 and/or anti-PD-1/PDL-1 immunotherapy is referred to as ICB associated colitis (36).

ICB-associated colitis acts as a distinct form of colitis that is characterized by flares reminiscent of those associated with IBD. Elucidation of the mechanisms involved in its immunopathogenesis will greatly enhance our understanding and therapeutic management of immune-mediated colitis (8). In our DSS model, we demonstrated that ICB treatment can aggregate the immunopathology of colitis. Our results with the B16 tumor model and DSS colitis are consistent with the clinical observations in patients who received ipilimumab (anti-CTLA-4 antibody), and/or nivolumab (anti-PD-1 antibody). The gut microbiota can drive the maturation and function of the immune system. Indeed, the bacterial microbiota profiling performed using 16S ribosomal RNA sequencing on the Illumina MiSeq platform revealed that the gut microbiota community structure, in particular, the microbial species present and their relative amounts, are associated with immunopathology of ICB-associated colitis. In our ICB-associated colitis model, the relative abundance of *Lactobacillus* was significantly lower in the stool of ICB-treated mice compared to the relative populations in the control and DSS administration only groups. Consistent with our finding, specific probiotic strains of *L. reuteri* were recently shown to suppress intestinal inflammation in a trinitrobenzene sulfonic acid (TNBS)-induced mouse colitis model (37). Taken together, these results suggest that some specific intestinal bacteria species may orchestrate the initiation of inflammation whereas other subsets may have a role in perpetuating ICB associated colitis.

In an effort to provide mechanistic evidence for the microbiota-ICB colitis connection, Wang et al. (19) recently administered a neutralizing anti-CTLA antibody to DSS-treated mice, and they found that compared with mice receiving the isotype-treatment, anti-CTLA-4 blockade led to more severe colitis that was further aggravated following pretreatment with the antibiotic vancomycin, suggesting a mitigating effect of the Gram-positive components of the microflora. In our study, we confirmed that administration of the probiotic *L. reuteri* could rescue the mice from the immunopathology of DSS-induced colitis. This rescue was accomplished without significant changes in the systemic or local numbers of T cells, predicting a minor or no effect on the antitumor effect of the immunotherapy. ILC3s are involved in the immunopathology of ICB-associated colitis. *L. reuteri* administration dramatically decreases the mucosal numbers of ILC3s. Interestingly, it is shown that tryptophan catabolites of *L. reuteri* have been recognized as ligands for aryl hydrocarbon receptor (AhR) (38). The introduction of *L. reuteri* was also shown to promote antitumor responses after PD-L1 blockade by augmenting dendritic cell function. Moreover, *L. reuteri* administration fully ameliorated the immunopathology of ICB-associated colitis without compromising the therapeutic efficacy of combined ICB against melanoma in our system.

Currently, many studies indicate a role for innate lymphoid cells (ILCs) in the pathogenesis of IBD (29, 39). Innate lymphoid cells (ILCs) are a recently discovered a group of innate immune cells. ILCs are detected in many organs and are especially enriched in the mucosal tissues of the human body. Some evidence has been provided to show that ILCs play crucial roles in the control of tissue homeostasis, act as effector cells in the immune responses to infections and function in inflammatory conditions (40). Indeed, several studies have reported that the ILC composition and function changes in the lamina propria of IBD patients (27, 41–44). In the majority of human studies, a potential role for ILCs was also found to be involved in Crohn's disease progression (29). The ILC component consists of three distinct groups: group 1 ILCs (ILC1s), group 2 ILCs (ILC2s), and group 3 ILCs (ILC3s). ILC3s have been shown to produce robust amounts of IL-22 and have important roles in maintaining the integrity of the intestinal barrier and promoting mucosal healing in IBD patients (45). In our DSS colitis model, the severity of the ICB-associated colitis was associated with increased mucosal numbers of group 3 innate lymphoid cells (ILC3s) and not with the levels of ILC1s and ILC2s, suggesting that ILC3s are the major innate lymphoid cell type involved in the immunopathology of ICB-associated colitis. *L. reuteri* administration dramatically decreased the mucosal numbers of ILC3s and therapeutically prevented colitis in the ICB-treated mice. Thus, it is likely that *L. reuteri* affects the immunopathology of ICB-associated colitis primarily by altering the local number of ILC3s. It should be noted that more research in this area is needed to clarify and integrate the current knowledge to improve treatment strategies for patients with ICB-associated colitis.

In summary, in this study, we provide strong evidence that modulation of the gut microbiome may abrogate ICB-associated colitis. If the same mechanisms are present in humans, this approach may provide a means to reduce or ameliorate the autoimmunity condition that often accompanies checkpoint blockade therapies, without diminishing the anticancer responses. This principle could apply to other checkpoint-related immunotherapies, such as the use of Tim3/LAG3-targeting antibodies or immunotherapy approaches directed against CAR-T cells and adoptive transfer.

## Data Availability

The 16S rRNA sequence data have been deposited in the NCBI BioProject with accession number PRJNA525923.

## Ethics Statement

The study protocol was reviewed and approved by the Institutional Review Board and Ethics Committee of Xinhua Hospital, Shanghai Jiao Tong University School of Medicine, China. All animal procedures were performed according to national guidelines and approved by the Animal Ethical and Experimental Committee of Xinhua Hospital, Shanghai Jiao Tong University School of Medicine.

## Author Contributions

LS and XY supervised the whole project, designed the experiments, analyzed data, and wrote the manuscript. TW performed the most experiments, analyzed data, and prepared the figures. NZ, QL, LJ, and BH contributed to some experiments and provided technical support. All authors read and approved the final manuscript.

### Conflict of Interest Statement

The authors declare that the research was conducted in the absence of any commercial or financial relationships that could be construed as a potential conflict of interest.

## Abbreviations

ICB: Immune Checkpoint Blockade
CTLA-4: cytotoxic-T-lymphocyte-associated protein 4
PD-1: programmed cell death protein 1 (PD-1)
PD-L1: programmed death-ligand 1
irAEs: immune-related adverse effects
DSS: dextran sulfate sodium
*L. reuteri*: Lactobacillus reuteri
ILC: innate lymphocytes.
