# Supplementary material for: Probiotics Lactobacillus reuteri Abrogates Immune Checkpoint Blockade-Associated Colitis by Inhibiting Group 3 Innate Lymphoid Cells
Source: Front Immunol. 2019 Jun 4;10:1235. doi: 10.3389/fimmu.2019.01235 (PMC6558076; doi:10.3389/fimmu.2019.01235)
Supplement: Supplementary file 1 [file Data_Sheet_1.docx]

Supplementary Material

**Probiotics *Lactobacillus reuteri* abrogates immune checkpoint blockade-associated colitis by inhibiting group 3 innate lymphoid cells**

Tingting Wang^1^, Naisheng Zheng^1^, Qin Luo^1^, Li Jiang^2^, Baokun He^3,4^, Xiangliang Yuan^1^*, Lisong Shen^1^*.

^1^Department of Clinical Laboratory, Xinhua Hospital, Shanghai Jiao Tong University School of Medicine, 200092 Shanghai, China.

^2^Department of Gynecology and Obstetrics, Xinhua Hospital, Shanghai Jiao Tong University School of Medicine, 200092 Shanghai, China.

^3^Department of Gastroenterology, Shanghai General Hospital, Shanghai Jiao Tong University School of Medicine, Shanghai 200080, China.

^4^Shanghai Key Laboratory of Pancreatic disease, Shanghai General Hospital, Shanghai Jiao Tong University School of Medicine, Shanghai, 201620, China.

*Correspondence to:

Lisong Shen, Email: lisongshen@hotmail.com;

or Xiangliang Yuan, Email: yuanxiangliang@gmail.com.

# Supplementary Figures and Tables

## Supplementary Table

**Supplementary Table S1. Antibody list for Flow cytometry.**

| **Name** | **Host** | **Clone** | **Company** | **Dilution** | **ref** |
| --- | --- | --- | --- | --- | --- |
| Fixable Viability Dye eFluor 450 | Mouse |  | eBioscience | 1:1000(FC) | 4338505 |
| Anti-mouse CD45 APC-Cy7 | Mouse | clone 30-F11 | Biolegend | 1:100(FC) | B230158 |
| Anti-mouse CD3 PerCP-Cy5.5 | Mouse | clone 17A2 | Biolegend | 1:100(FC) | B233420 |
| Anti-mouse CD3 PE-Cy7 | Mouse | clone 145-2C11 | BD | 1:100(FC) | 8019605 |
| Anti-mouse CD4 FITC | Mouse | clone GK1.5 | eBioscience | 1:200(FC) | 4313007 |
| Anti-mouse CD8a PE | Mouse | clone 53-6.7 | eBioscience | 1:200(FC) | 4300680 |
| Anti-mouse GR-1 PE-Cy7 | Mouse | clone 1A8 | BD | 1:100(FC) | 8096641 |
| Anti-mouse CD19 PE-Cy7 | Mouse | clone 1D3 | BD | 1:100(FC) | 8030704 |
| Anti-Mouse CD127 (IL7Ra) PerCP-Cy5.5 | Mouse | clone A7R34 | Biolegend | 1:100(FC) | B249756 |
| Anti-Mouse RORγt PE | Mouse | clone Q31-378 | BD | 1:100(FC) | 7201863 |
| Anti-mouse NKp46 APC | Mouse | clone 29A1.4 | Biolegend | 1:100(FC) | B252578 |
| Anti-mouse KLRG-1 FITC | Mouse | clone 2F1/KLRG1 | Biolegend | 1:100(FC) | B252432 |
| Anti-Mouse CD11b PE-Cy7 | Mouse | clone M1/70 | Biolegend | 1:100(FC) | B203625 |
| Anti-Mouse Ly-6G PerCP-Cy5.5 | Mouse | clone RB6-8C5 | eBioscience | 1:200(FC) | 4310362 |
| Anti-Mouse ki67 APC | Mouse | clone SolA15 | eBioscience | 1:200(FC) | 4342181 |
| Foxp3 / Transcription Factor Staining Buffer Set | Mouse |  | eBioscience |  | 4343791 |

## Supplementary Figures

**
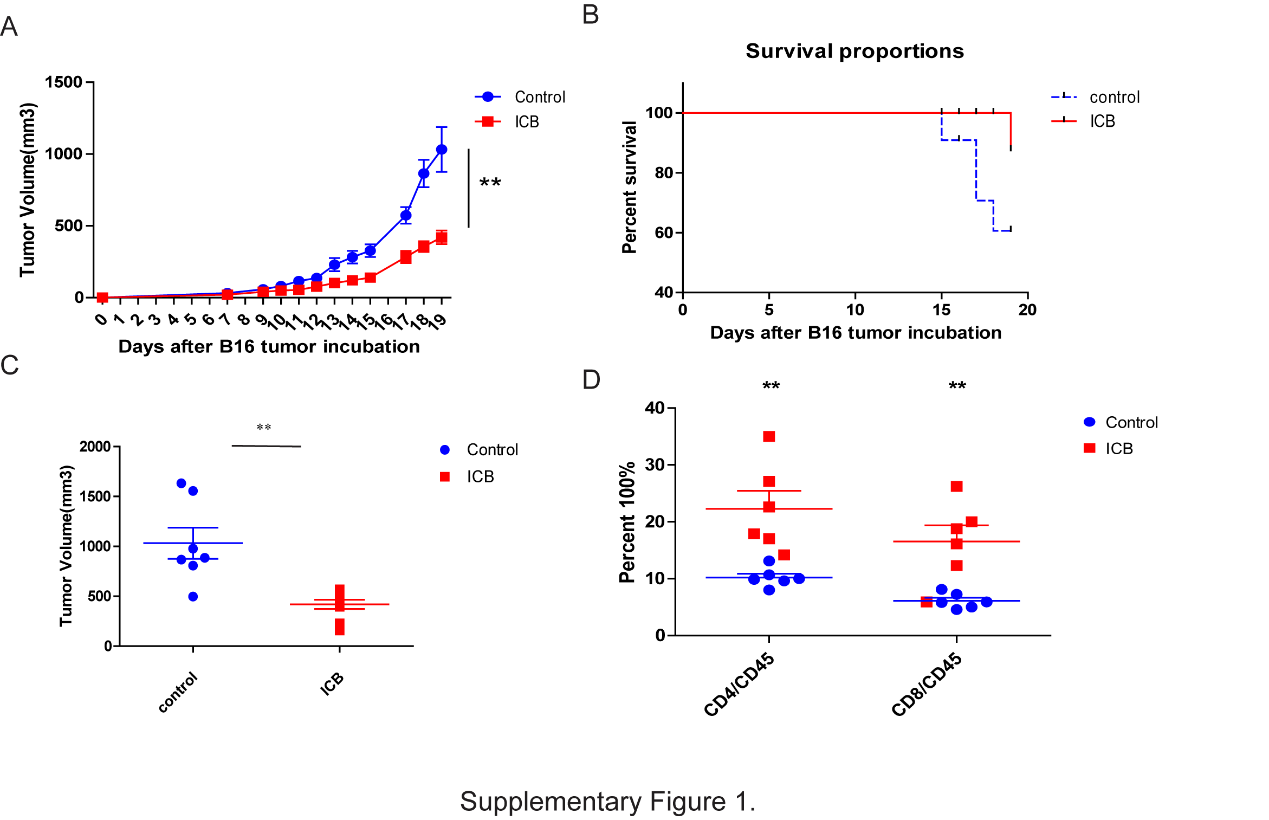
**

**Supplementary Figure S1. Immune checkpoint blockades induce tumor regression and increase T cell infiltration in the B16 melanoma model**

**(A),** Impact of ICB (anti-CTLA4 Ab and anti-PD1 Ab) treatment on B16 tumor volume. **(B),** The survival curve of mice bearing B16 tumor with the administration of isotype control Abs or ICB. (**C**). Tumor sizes of the B16 tumors on day 19 postimplantation in mice treated with Ctrl or ICB. (**D**). Quantification of intratumoral CD4^+^ T cells and CD8^+^ T cells in B16 melanoma tumor with indicated treatment by flow cytometry (19 days of post tumor implantation; n=5 per group).

**
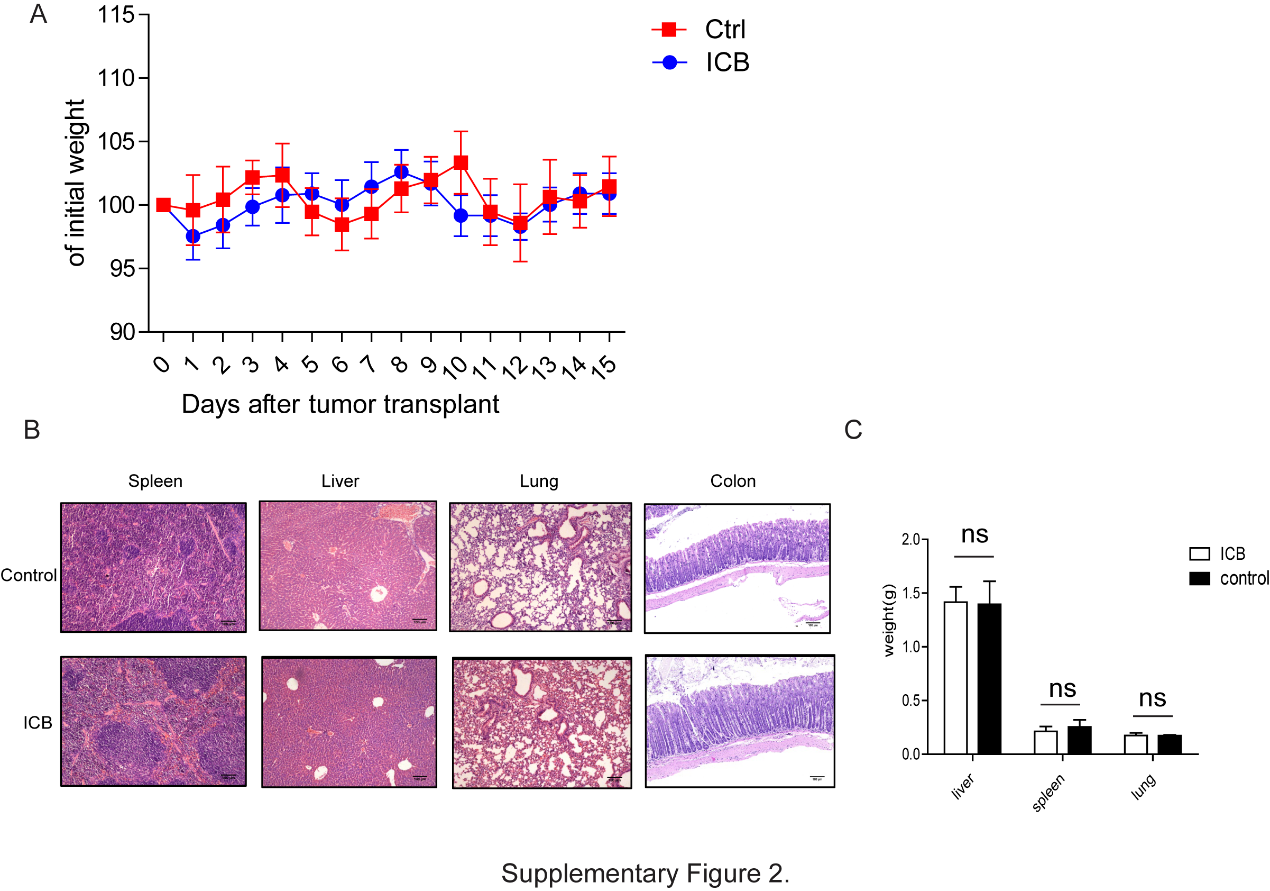
**

**Supplementary Figure S2. The toxicity of ICB on B16 tumor-bearing mice. (A),** Weight curves of mice receiving the immune checkpoint blockade (ICB: αCTLA-4 mAb and αPD-1 mAb) or IgG isotype control (Ctrl). n = 5 per group. **(B)**, Representative spleen, liver, lung and colon histological results from mice treated with an injection of the isotype control (upper) or ICB treatment (down). Tissue samples were collected on day 15 and stained with H&E. (Scale bar, 100μm.). **(C)**, the weight of indicated tissue (liver, spleen and lung) mice treated with an injection of the isotype control or ICB treatment. Tissue samples were collected on day 15. n.s., not significant.

**
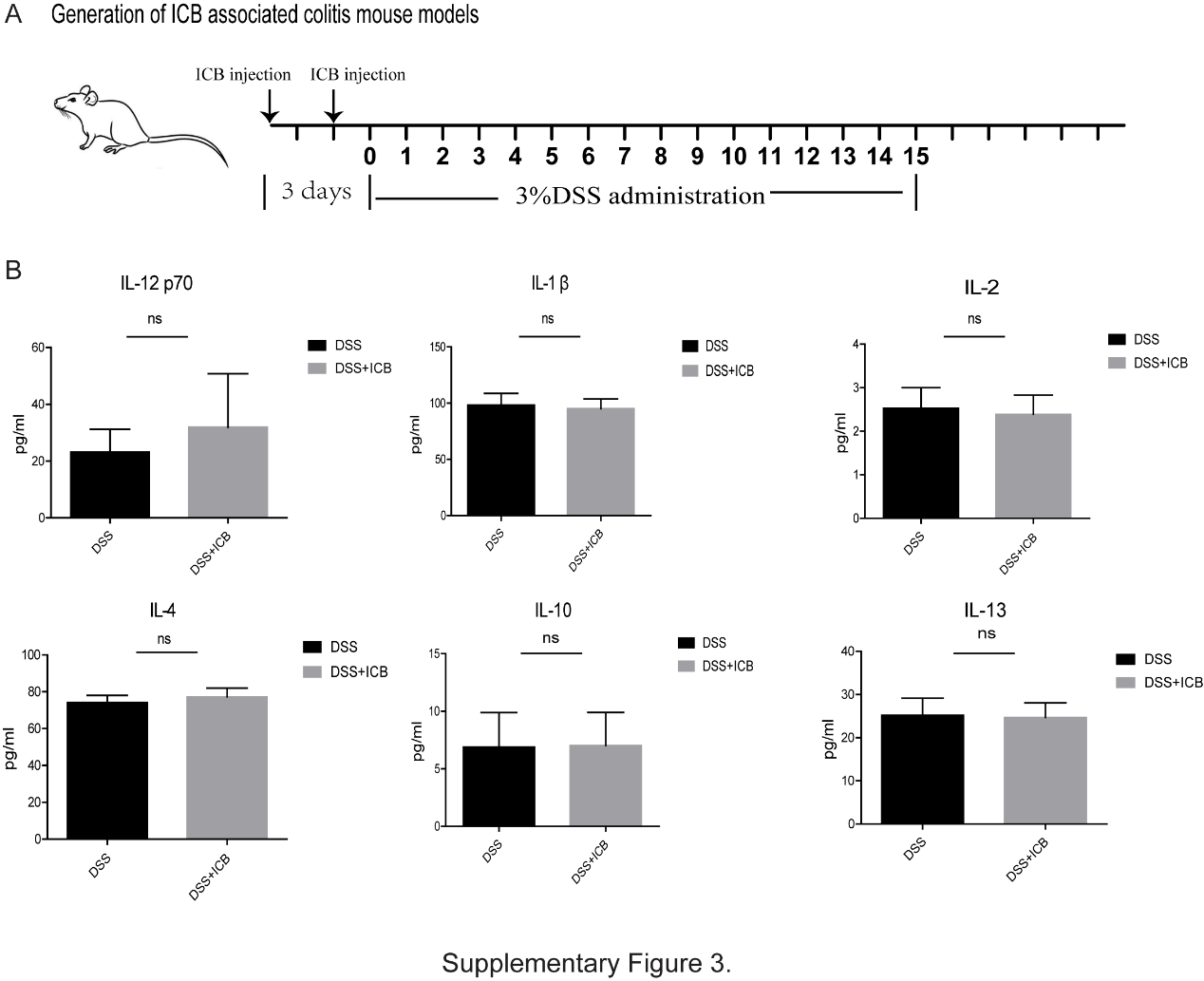
**

**Supplementary Figure S3. Cytokine profile in DSS-induced mice with isotype control or ICB treatment.** (**A**). the diagram shows the generation strategy of ICB associated colitis model. (**B**) The IL12p70, IL1-β, IL-2, IL-4, IL-10, and IL-13 concentrations in the sera of mice treated with isotype control or the ICB treatment along with 7 d of 3% DSS administration, n = 5 per group. Means with SEM analyzed by unpaired Student’s t-test. n.s., not significant.

**
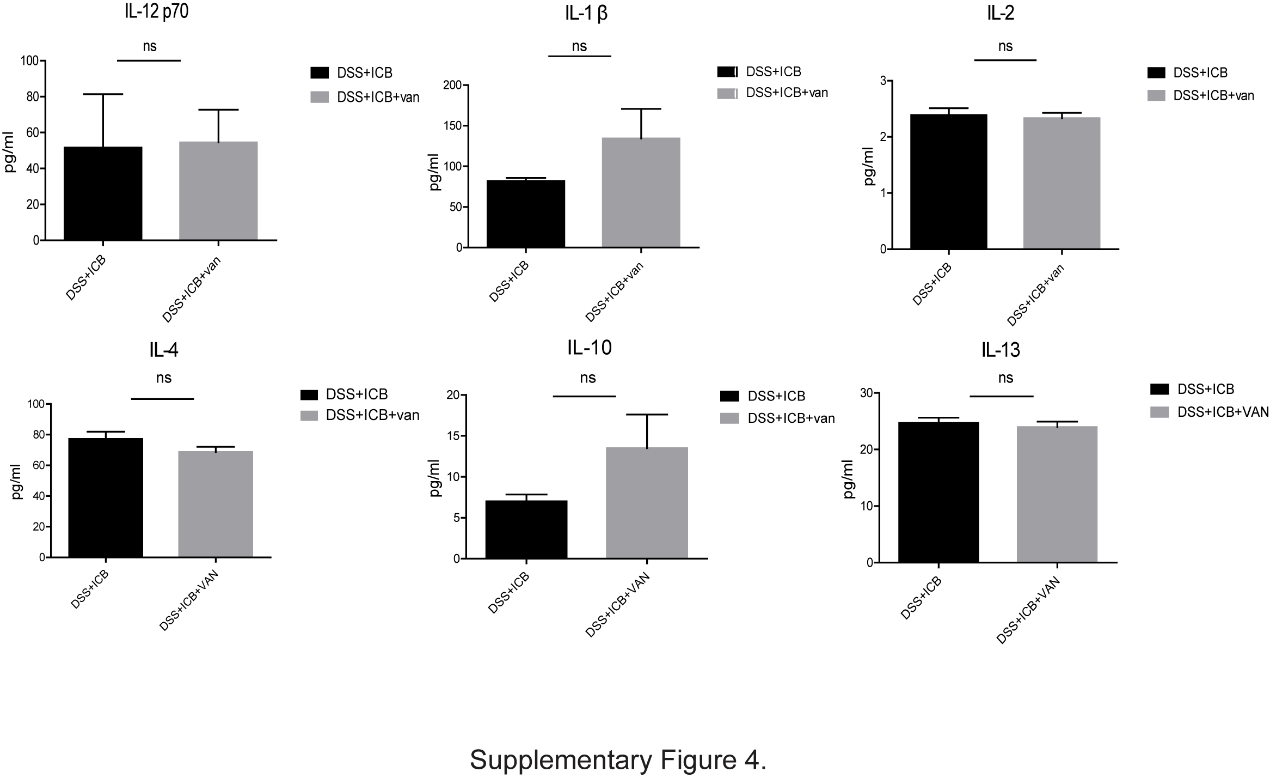
**

**Supplementary Figure S4. Cytokine profile in DSS-induced mice with ICB or ICB + Vancomycin treatment.** The IL12p70, IL1-β, IL-2, IL-4, IL-10, and IL-13 concentrations in the sera of mice in the sera of water- or vancomycin-treated mice with the indicated treatments. n = 5 per group. Means with SEM analyzed by unpaired Student’s t-test. n.s., not significant.


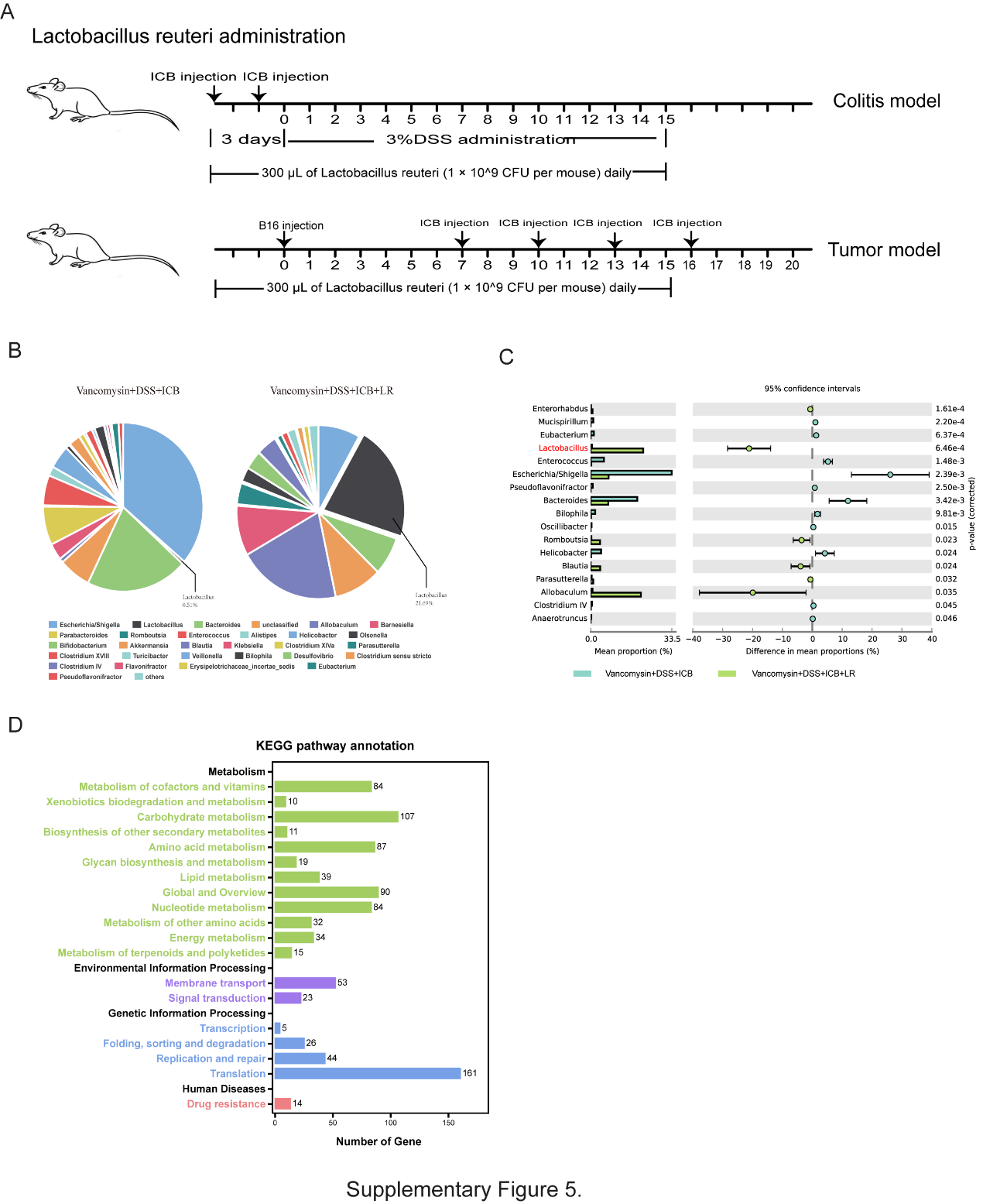


**Supplementary Figure S5. The differential abundance analysis and KEGG pathway annotation of *L. reuteri* in mice with indicated treatment.**

(**A**). the diagram shows the strategy of colitis model and B16 melanoma tumor model with the administration of *L. reuteri*. (**B**) the composition of the gut microbiome in ICB-injected mice with 3% DSS-induced colitis treated either with vancomycin + PBS, or vancomycin + *L. reuteri*, n = 5 per group. (**C**). The differential abundance analysis of enriched predominant bacteria in ICB-injected mice with 3% DSS-induced colitis treated either with vancomycin + PBS, or vancomycin + *L. reuteri*, n = 5 per group. Welch’s t-test is used. The p value was obtained by the test is subjected to multiple test correction using FDR to obtain a p value. *P* value <0.05, indicating significant difference. **(D)**. Kyoto Encyclopedia of Genes and Genomes (KEGG) pathway analysis of *L. reuteri* in ICB-injected mice with 3% DSS-induced colitis treated with vancomycin + *L. reuteri*.


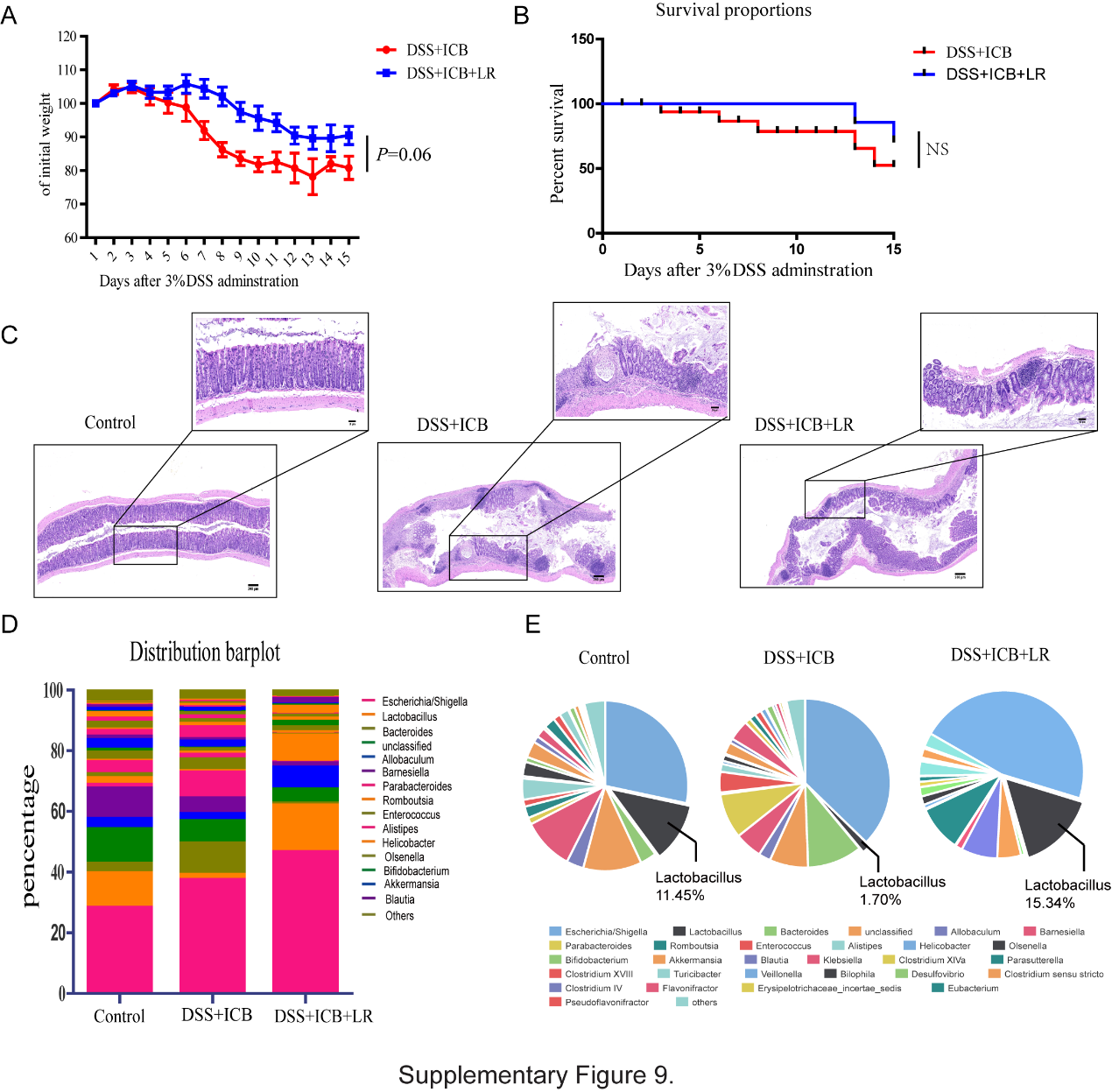


**Supplementary Figure S6. *L. reuteri*** **therapeutically abrogates ICB-associated intestinal inflammation in DSS-treated mice.** (A). Weight loss curves of ICB-injected mice with 3% DSS-induced colitis treated either with PBS, or *L. reuteri*, n = 15 per group. (**B).** Percent survival curves of in ICB-injected mice with 3% DSS-induced colitis treated as described in A. Survival was monitored for 14 d, n = 15 per group. NS: no significant. (**C)**. Representative colonic histology results from ICB-injected mice with 3% DSS-induced colitis with the indicated treatments. Colon samples were collected on day 10 and H&E stained. (Scale bar, 200 μm). (D). Microbial community bar plot sorted by the genus of the mice receiving ICB treatment and 3% DSS administration with or without ICB. The relative abundances of the predominant bacteria (>1% in any sample) in the feces of mice receiving the ICB treatment along with 3% DSS administration. n = 5 per group. (E). The pie chart shows the relative abundances of the predominant bacteria (>1% in any sample) in the feces of mice treated with 3% DSS administration and with or without ICB. n = 5 per group.

**
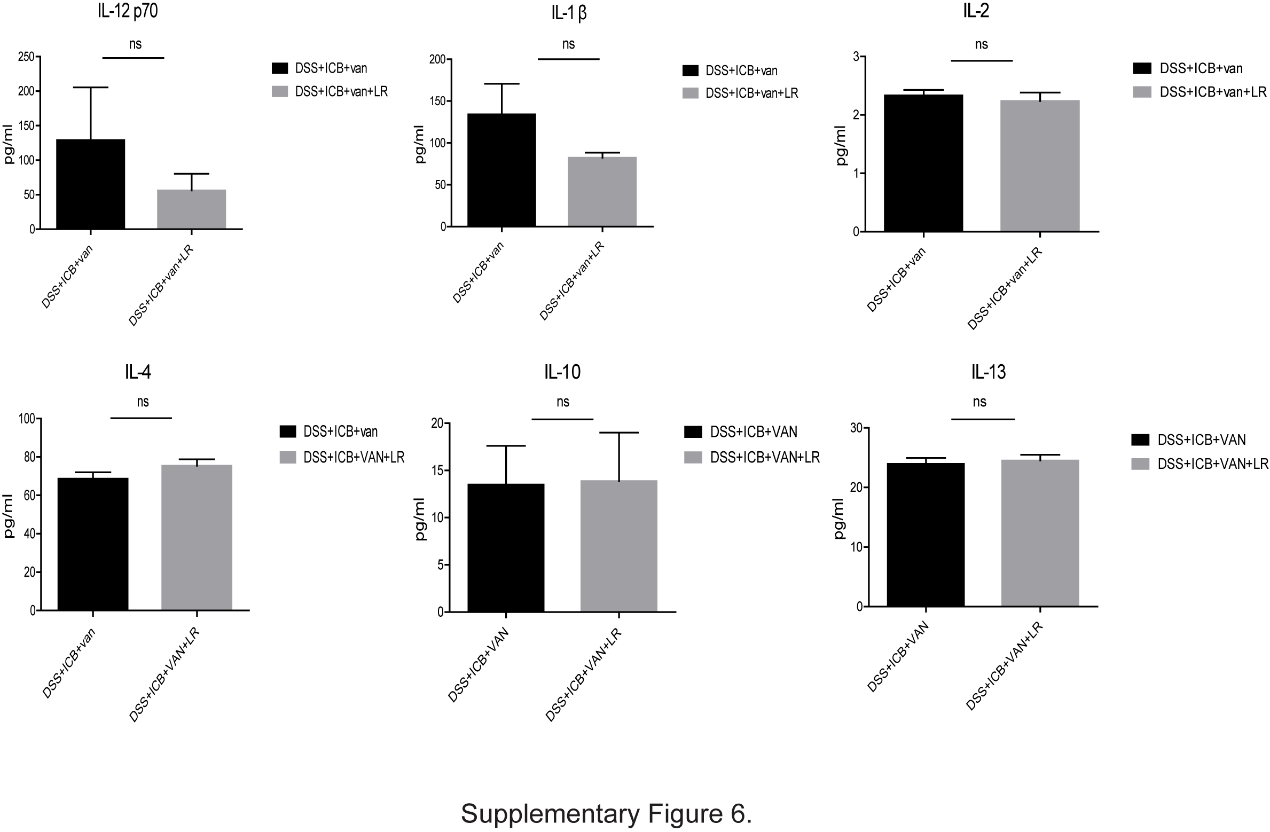
**

**Supplementary Figure S7. Cytokine profile in ICB-injected mice with 3% DSS-induced colitis treated with vancomycin + PBS or vancomycin + *L. reuteri*.** The IL12p70, IL1-β, IL-2, IL-4, IL-10, and IL-13 concentrations in the sera of mice with the indicated treatments. n = 5 per group. Means with SEM analyzed by unpaired Student’s t-test. n.s., not significant.

**
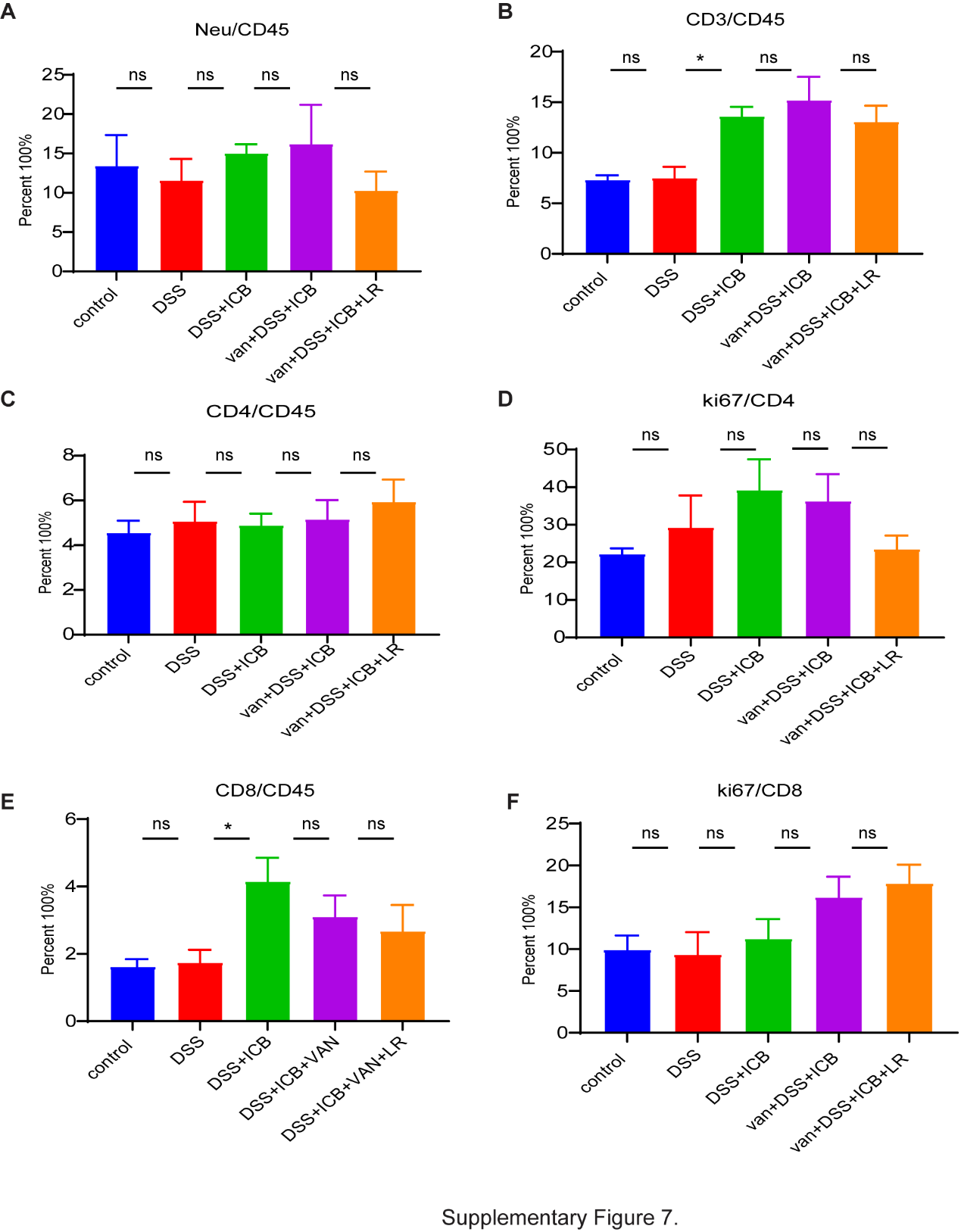
**

**Supplementary Figure S8. Flow cytometric analysis of immune cells in intestinal lamina propria of mice receiving indicated treatment.** Quantification of the immune cells, neutrophils (A), CD3+ T cells (B), CD4+CD3+ T cells (C), Ki67+ CD4+ T cells (D), CD8+ T cells (E), and Ki67+ CD4+ T cells (F) isolated from the large intestinal lamina propria of control mice or mice receiving the IgG isotype control (Iso Ctrl), ICB, ICB+Van, and ICB+Van+ L. reuteri (LR) treatment along with 3% DSS administration.

**
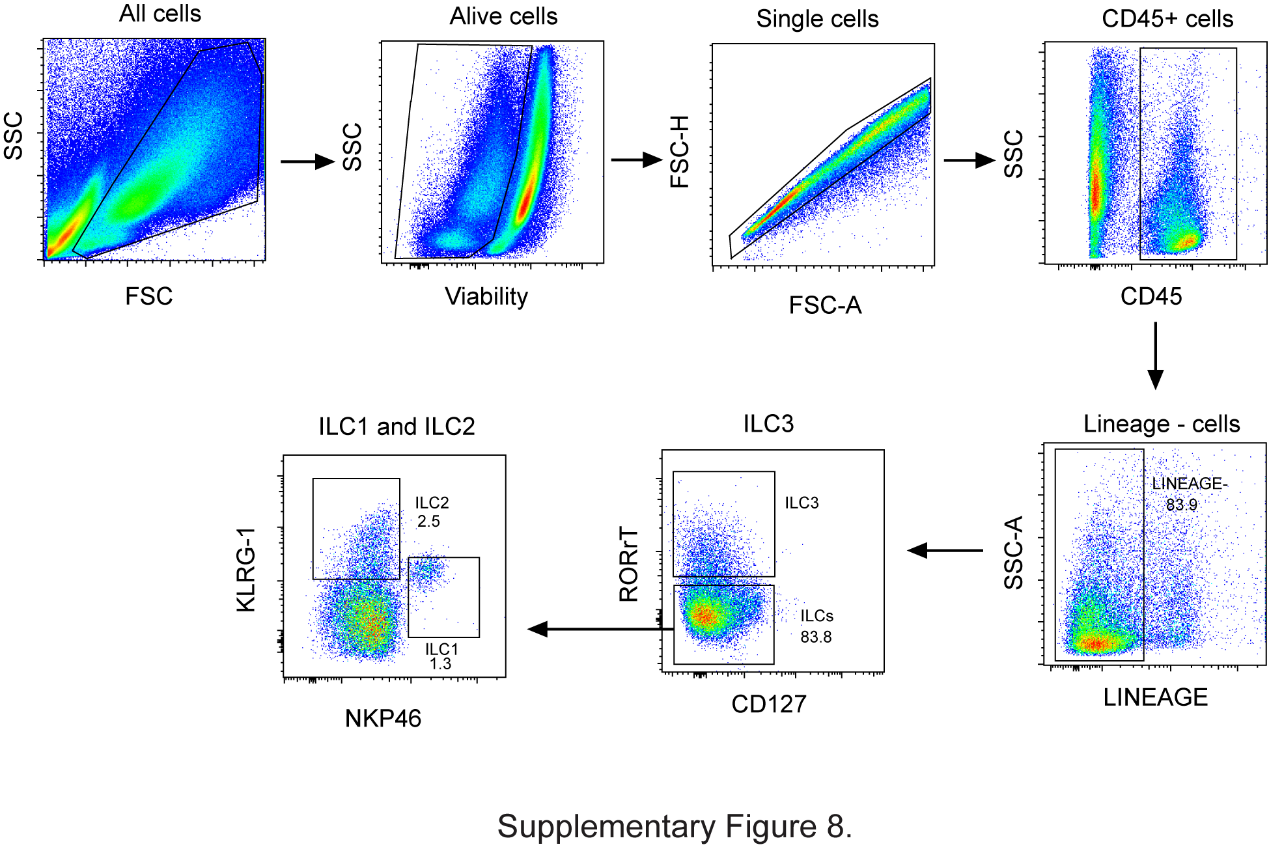
**

**Supplementary Figure S9. The flow cytometry gating strategies for analyzing canonical ILC subtypes**. Initial gating was on immune cells as approximated by scattering, then dead cells and doublets were eliminated. Next, CD45^+^ cells were selected, lineage (CD3/CD19/Gr1) negative cells were gated for total innate lymphoid cells, and then analyzed the RORrt+ cells as canonical ILC3, CD127^low^ RORrt- cells were separated to NKP46+KLRG1- ILC1 and NKP46-KLRG1+ ILC2.

**
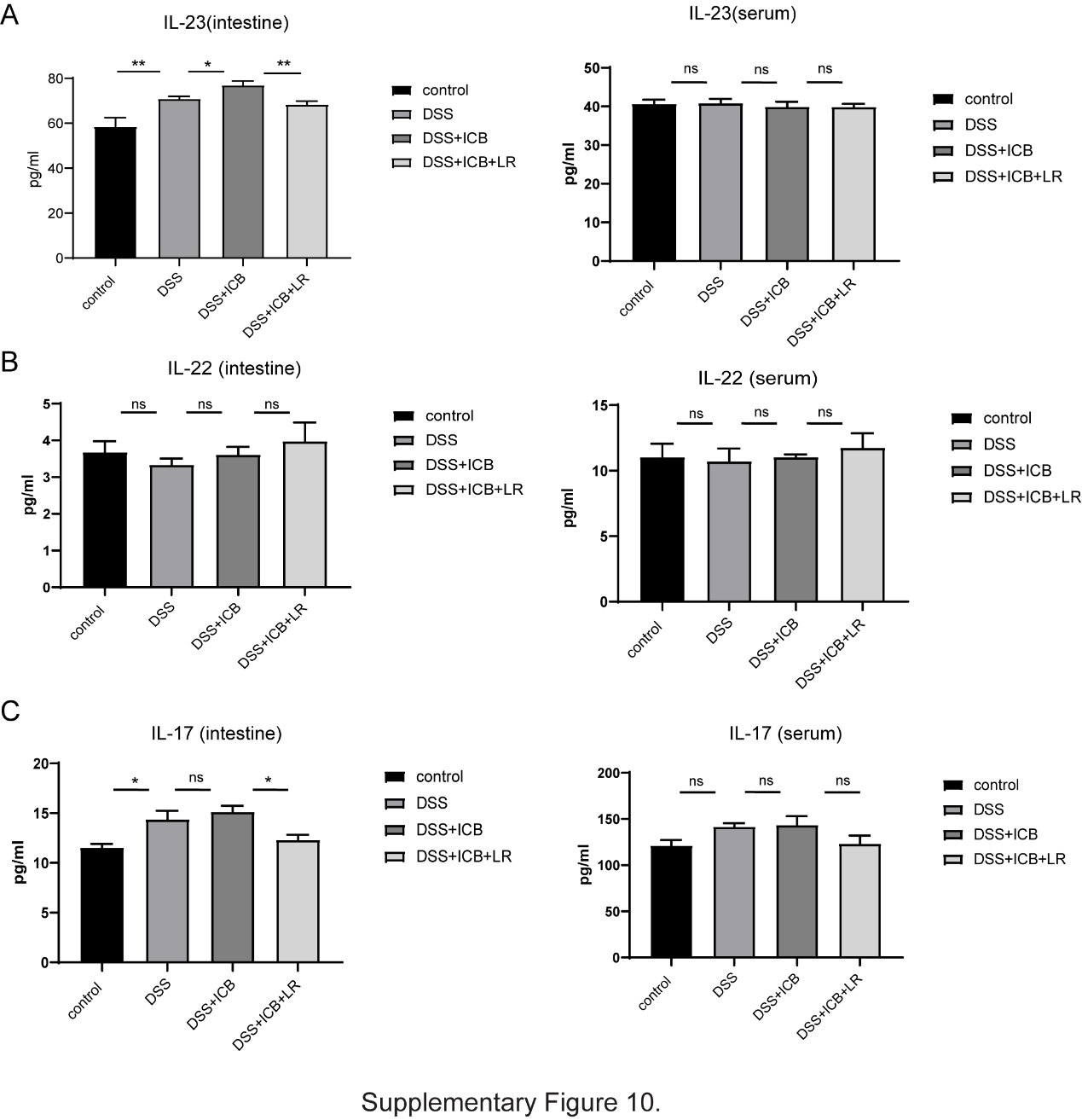
**

**Supplementary Figure S10. Cytokine profile in ICB-injected mice with DSS induced colitis treated with or without *L. reuteri*.** The IL-23 (A), IL-22 (B), and IL-17 (C) concentrations in the intestine tissue or sera of mice with the indicated treatments. n = 5 per group. Means with SEM analyzed by unpaired Student’s t-test. *, *P*<0.05; n.s., not significant.
